# Evaluating the quality and reliability of Kawasaki disease–related content on TikTok and Bilibili: a cross-sectional study

**DOI:** 10.3389/fpubh.2025.1664542

**Published:** 2026-01-22

**Authors:** Nan Shen, Chang Xu, Yongkun Yang, Dongli Zhang

**Affiliations:** 1 Jiangyin People’s Hospital, Wuxi, Jiangsu, China; 2 The Affiliated Taizhou People's Hospital of Nanjing Medical University, Taizhou, Jiangsu, China

**Keywords:** Bilibili, health education, Kawasaki disease, quality assessment, TikTok

## Abstract

**Background:**

As public interest in health and immunology grows, short video platforms have become an increasingly important source of medical information. Kawasaki disease, a pediatric immune-mediated vasculitis with potential cardiovascular complications, has attracted substantial attention; however, the accuracy and quality of related content on these platforms remain unexamined. This study aimed to evaluate the overall quality of Kawasaki disease–related videos on TikTok and Bilibili.

**Method:**

On February 25, 2025, newly registered accounts were used to search the term “川崎病” (Kawasaki disease) on TikTok and Bilibili, and the top 100 videos from each platform were collected. Video quality was evaluated using the JAMA benchmark criteria, a modified DISCERN, and PEMAT, while user engagement metrics (likes, comments, saves, and shares) were analyzed for correlations.

**Results:**

A total of 146 videos were included. Although TikTok videos demonstrated higher quality and popularity than those on Bilibili, overall video quality on both platforms remained suboptimal. Median JAMA scores were 2.00 and 1.00, modified DISCERN scores were both 3.00, intelligibility was 70% versus 64%, and operability was 67% on both platforms. Most videos were monologue-based and symptom-focused, with pediatricians and individual users as the main uploaders. Pediatricians and individual users were the two largest groups of content creators. Pediatrician-uploaded videos showed higher quality and engagement, whereas individual-user videos were more often misleading and less interactive. Five major misinformation themes were identified, including symptom oversimplification, incorrect etiological claims, promotion of non–evidence-based home treatments, misunderstanding of diagnostic criteria, and misleading statements about immunoglobulin therapy. Video quality was positively correlated with popularity, while longer duration was negatively associated with both quality and engagement. Heterogeneity was observed across platforms.

**Conclusion:**

The quality and reliability of Kawasaki disease–related videos on short video platforms remain suboptimal, highlighting the need to address misinformation, refine evaluation tools, and promote high-quality content creation.

## 1 Introduction

Kawasaki disease, also known as mucocutaneous lymph node syndrome, is an acute febrile systemic vasculitis of unknown etiology that primarily affects children under 5 years of age (1). It has become a significant global public health concern due to its potential to cause serious cardiovascular complications (2). It is characterized by prolonged fever, mucocutaneous inflammation, lymphadenopathy, and coronary artery abnormalities, and is the leading cause of acquired heart disease in children in industrialized countries (3). Epidemiological data indicate a rising incidence of Kawasaki disease over recent decades (4). Without timely treatment, approximately 25% of affected children may develop coronary artery aneurysms, which can lead to myocardial infarction or sudden death in severe cases (5). Early recognition and understanding of Kawasaki disease are therefore crucial for prompt diagnosis and intervention. In this context, public self-education plays an essential role in increasing disease awareness and promoting early medical consultation (6).

In recent years, social media platforms have become a major source of health information for the public (7), with short video applications such as TikTok and Bilibili gaining widespread popularity across different populations. TikTok, a short video-sharing platform, leverages algorithmic recommendations and engaging content formats to achieve high user activity and influence, particularly among younger audiences (8). The app boasts over 2 billion users across more than 150 countries and regions, with over 200 million downloads in the United States alone (9). Bilibili, a prominent Chinese bullet-screen video-sharing platform, has an average of 340 million monthly active users. Its extensive library of high-quality videos has attracted a large, predominantly young user base, making it an emerging channel for health information dissemination in China (10). These platforms have facilitated public health education by delivering content in a concise and appealing manner, making it more accessible and acceptable to a wider audience. Recent studies have supported a notable increase in the dissemination of health-related information via social media platforms over the past decade (11), with growing public reliance on these platforms for health education and public health awareness (12). This highlights the significant potential of social media in health communication.

However, the quality, accuracy, and reliability of health-related content on these platforms vary considerably and often lack professional oversight. Concerns about misinformation remain prevalent (13). Previous studies have evaluated the quality and reliability of social media videos related to Attention-Deficit/Hyperactivity Disorder (14), gastric cancer (15), and colorectal polyps (7), revealing frequent deficiencies or misleading information. Despite these efforts, the quality of social media content regarding other pediatric conditions remains underexplored. In particular, Kawasaki disease—an important clinical concern in pediatrics—has received limited attention in terms of its health communication on social media platforms. To the best of our knowledge, no study has yet systematically assessed the quality and reliability of Kawasaki disease–related content on short video platforms.

Given the public’s growing reliance on medical information from social media, there is an urgent need to systematically evaluate the quality and reliability of Kawasaki disease–related videos on TikTok and Bilibili. Understanding the current state of such content is crucial for identifying potentially misleading information and guiding the public toward authoritative health education resources.

## 2 Materials and methods

### 2.1 Search strategy and data collection

**Figure 1:**
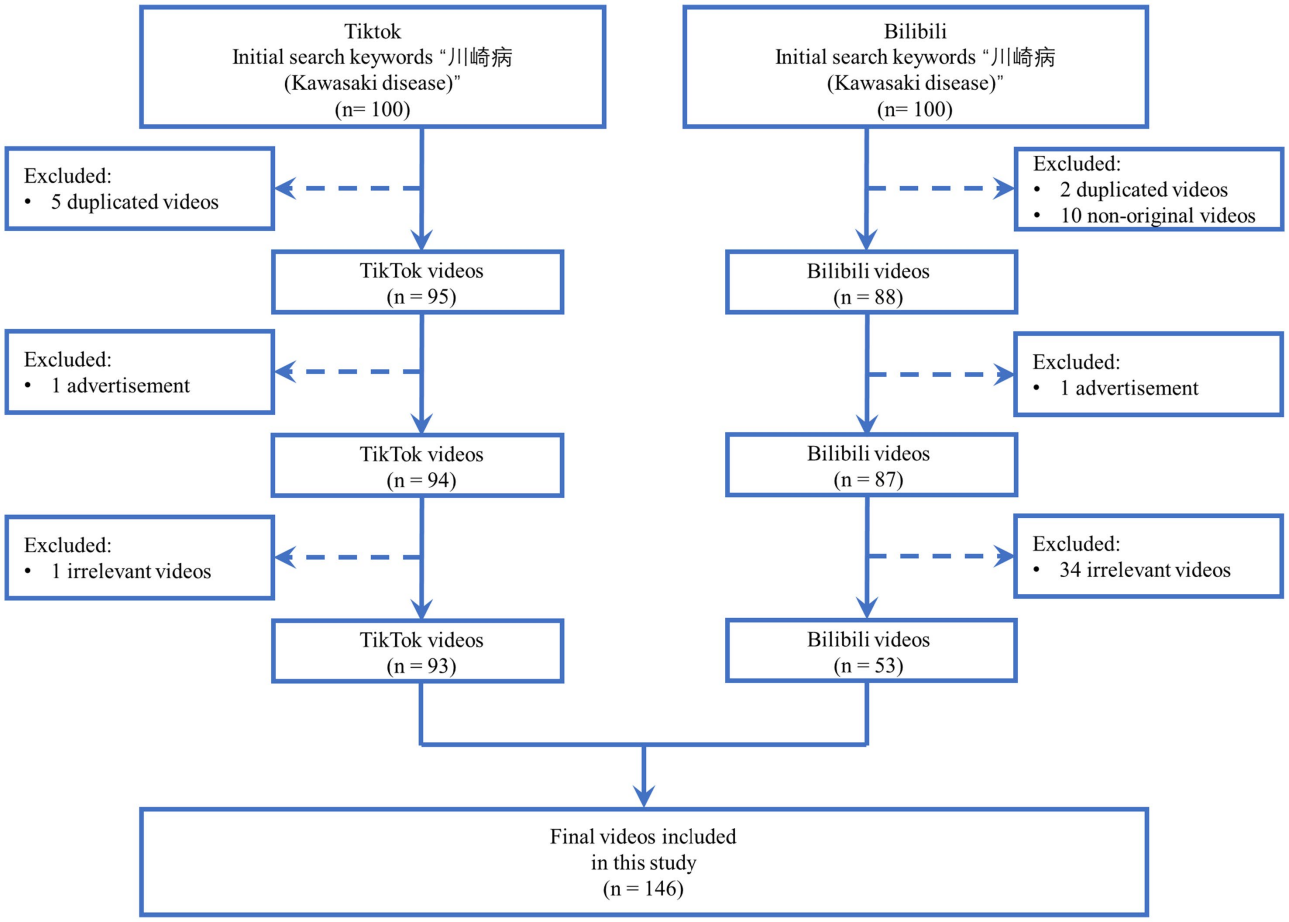
Flow diagram of video screening and inclusion.

This study is a cross-sectional content analysis conducted on February 25, 2025. We used the keyword “川崎病” (Kawasaki disease) to search the Chinese versions of TikTok and Bilibili (see Figure 1). To minimize bias caused by personalized recommendation algorithms, new accounts were registered and logged in on each platform prior to searching. All videos were collected and downloaded by one researcher (Nan Shen). On each platform, the top 100 videos from the search results were retrieved, based on the assumption that users typically do not watch more than 100 short videos per day (16).

Inclusion criteria were as follows: (1) the video content was related to Kawasaki disease, and (2) the video was in Chinese. Exclusion criteria included: (1) duplicate content; (2) promotional or advertisement-related content; (3) videos unrelated to Kawasaki disease; and (4) non-original videos.

For all videos that met the inclusion criteria, the following characteristics were recorded and analyzed: uploader name, uploader category, number of likes, saves, comments, and shares, upload date, video duration, presentation format, and main content theme.

### 2.2 Video classification

Based on the source of the videos, uploaders were categorized into five groups: pediatricians, doctor (non-pediatrician), medical institution, personal, and official media outlets. Video content was classified into the following thematic categories: epidemiology, etiology, symptoms, diagnosis, treatment, prevention, prognosis, follow-up, and nursing. The presentation formats of the videos were categorized as follows: personal monologue, Q&A format, PowerPoint explanation, animation, medical scene, documentary style, and other formats. Detailed classifications are presented in Supplementary Table S1.

### 2.3 Assessment of video quality and reliability

To assess the quality and reliability of the videos, we employed three evaluation tools: the Journal of the American Medical Association (JAMA) benchmark criteria, the modified DISCERN tool, and the Patient Education Materials Assessment Tool (PEMAT). The JAMA benchmark criteria evaluate four dimensions—authorship, attribution, disclosure, and currency—to determine the academic rigor and credibility of the content (17). The modified DISCERN tool, widely used to assess the quality of health information, consists of five questions that primarily evaluate the reliability of health-related content, especially regarding treatment options (18). The PEMAT tool assesses the intelligibility and operability of patient education materials, aiming to determine how easy the content is to comprehend and apply. Higher scores indicate better educational effectiveness (19). Detailed evaluation items for each scoring tool are provided in Supplementary Tables S2–S4.

Additionally, we conducted a systematic qualitative thematic analysis of videos uploaded by individual users that contained inaccurate information. Each individual video was allowed to be assigned to multiple themes. The analytic process followed Colaizzi’s seven-step method: (1) familiarization with the data; (2) identification of significant statements; (3) formulation of meaning units; (4) clustering of themes; (5) comprehensive description; (6) development of the fundamental structure; and (7) validation of the fundamental structure (20).

Prior to formal scoring, two evaluators (Nan Shen and Chang Xu) jointly reviewed the scoring criteria for the JAMA, modified DISCERN, and PEMAT tools and discussed specific details to minimize cognitive bias. Subsequently, all videos were independently assessed by the two researchers (Nan Shen and Chang Xu). In cases of uncertainty, consensus was reached through discussion involving a third reviewer (Yongkun Yang). To ensure comprehensive evaluation and accuracy, each video was scored twice by Nan Shen and once by Chang Xu.

We used the intraclass correlation coefficient (ICC) to assess inter-rater agreement, which was also applied in this study to evaluate the reliability of each scoring tool. The interpretation of reliability was as follows: poor (ICC < 0.50), moderate (ICC = 0.50–0.75), good (ICC = 0.75–0.90), and excellent (ICC > 0.90) (21). The ICC values for the two raters across different tools were as follows: JAMA score (ICC = 0.89, 95% CI: 0.86–0.92), modified DISCERN score (ICC = 0.72, 95% CI: 0.69–0.75), intelligibility (ICC = 0.85, 95% CI: 0.83–0.88), and operability (ICC = 0.83, 95% CI: 0.81–0.85). Intra-rater reliability showed strong performance: JAMA score (0.96), modified DISCERN score (0.76), intelligibility (0.80), and operability (0.84).

### 2.4 Statistical analysis

Descriptive analyses were conducted to summarize the data. As the continuous variables did not follow a normal distribution, they were presented as median with interquartile range (IQR). Categorical variables were described using frequencies and percentages. The Kruskal-Wallis test was used to compare video quality and reliability scores across multiple groups, followed by Dunn’s *post-hoc* test for pairwise comparisons. Bonferroni correction was applied to adjust for the risk of type I error due to multiple comparisons. Spearman correlation analysis was performed to assess associations between video engagement metrics (e.g., number of likes, comments, shares, duration) and quality/reliability scores. A Poisson regression model was used to evaluate the influence of video characteristics (including uploader type, content category, and engagement metrics) on quality scores. All statistical tests were two-sided, and a *p*-value < 0.05 was considered statistically significant. Analyses were conducted using R version 4.3.0, with data processing and visualization performed using R packages such as tidyverse, ggplot2, and rstatix.

Platform-specific analyses were additionally conducted for TikTok and Bilibili, with detailed results provided in the Supplementary materials.

### 2.5 Human ethics and consent to participate declarations

This study did not involve any clinical data, human participants, or experimental animals. All data were obtained from publicly available videos on the TikTok and Bilibili platforms, without involving any user privacy information or direct interaction with users. Therefore, ethical approval was not required for this study. The authors have carefully read and complied with the Terms of Use of both TikTok and Bilibili.

## 3 Results

### 3.1 General characteristics of the videos

After excluding duplicate, advertising, irrelevant, and non-original videos, a total of 53 Bilibili and 93 TikTok videos were included for further data extraction and analysis (Figure 1).

Table 1 lists the general characteristics of the videos. Our study found that TikTok videos received more likes, comments, shares, and saves than Bilibili videos (*p* < 0.001), while Bilibili videos were longer in duration than TikTok videos (*p* < 0.001). In terms of video quality evaluation, TikTok videos had significantly higher JAMA scores compared to Bilibili videos (*p* < 0.001). Furthermore, TikTok videos were rated higher for intelligibility than Bilibili videos, with a statistically significant difference (*p* = 0.004), while there was no significant difference in operability between the two platforms.

**Table 1:** General characteristics and scoring of Kawasaki disease–related videos.

Variable	Overall (*N* = 146)	Bilibili (*n* = 53)	Tiktok (*n* = 93)	*p*-value
Likes, median (p25–p75)	107.50 (27.00–568.00)	17.00 (5.00–46.00)	355.00 (78.00–2,035.00)	<0.001
Comments, median (p25–p75)	16.50 (2.00–148.00)	1.00 (0.00–5.00)	63.00 (10.00–312.00)	<0.001
Shares, median (p25–p75)	70.00 (15.00–447.00)	12.00 (3.00–25.00)	264.00 (50.00–1,036.00)	<0.001
Saves, median (p25–p75)	50.50 (15.00–275.00)	22.00 (6.00–51.00)	110.00 (27.00–727.00)	<0.001
Time, median (p25–p75)	104.50 (55.00–256.00)	299.00 (150.00–1,231.00)	79.00 (46.00–121.00)	<0.001
JAMA, median (p25–p75)	2.00 (1.00–2.00)	1.00 (1.00–1.00)	2.00 (2.00–2.00)	<0.001
DISCERN, median (p25–p75)	3.00 (3.00–3.00)	3.00 (2.00–3.00)	3.00 (3.00–3.00)	0.025
Intelligibility, median (p25–p75)	70.00 (62.00–70.00)	64.00 (60.00–70.00)	70.00 (62.00–70.00)	0.004
Operability, median (p25–p75)	67.00 (67.00–100.00)	67.00 (67.00–75.00)	67.00 (67.00–100.00)	0.055

### 3.2 Video source, presentation format, and content

Overall, pediatricians and individual users uploaded a comparable proportion of videos (57/146, 39.04% vs. 48/146, 32.88%). Specifically, on TikTok, pediatricians uploaded the most videos, accounting for 52.69% (49/93), followed by individual users (17/93, 18.28%), official media (11/93, 11.83%), medical institutions (9/93, 9.68%), and non-pediatric doctors (7/93, 7.53%). On Bilibili, individual users uploaded the most videos (31/53, 58.49%), followed by pediatricians and non-pediatric doctors (8/53, 15.09%), medical institutions (6/53, 11.32%), with no videos uploaded by official media.

Monologues dominated the video format, accounting for 54.11% (79/146) of the total. Regarding video types on TikTok, the majority were monologues (72.04%, 67/93), followed by medical scenes (21.51%, 20/93), with animations, PowerPoint presentations, and Q&A formats comprising only 6.46% (6/93) in total. On Bilibili, PowerPoint presentations were the most common (45.28%, 24/53), followed by monologues (22.64%, 12/53), medical scenes (11.32%, 6/53), and other formats including animations, documentaries, Q&A, and others.

The content of the videos predominantly focused on disease symptoms (124/282, 43.97%) and treatment (56/282, 19.86%). Similarly, the content related to symptoms in TikTok and Bilibili accounted for 48.47% (79/163) and 34.88% (45/129), respectively, followed by treatment (TikTok: 27/163, 16.56%; Bilibili: 29/129, 22.48%) and diagnosis (TikTok: 19/163, 11.66%; Bilibili: 19/129, 14.73%) (Table 2).

**Table 2:** Content characteristics of Kawasaki disease–related videos.

Variable	Overall, *n* (%)	Bilibili, *n* (%)	Tiktok, *n* (%)	p-value
Video source (*N* = 146)				<0.001
Medical institution	15.00 (10.27%)	6.00 (11.32%)	9.00 (9.68%)	
Doctor (non-pediatrician)	15.00 (10.27%)	8.00 (15.09%)	7.00 (7.53%)	
Official media	11.00 (7.53%)	0.00 (0.00%)	11.00 (11.83%)	
Personal	48.00 (32.88%)	31.00 (58.49%)	17.00 (18.28%)	
Pediatrician	57.00 (39.04%)	8.00 (15.09%)	49.00 (52.69%)	
Video presentation format (*N* = 146)				<0.001
Animation	6.00 (4.11%)	4.00 (7.55%)	2.00 (2.15%)	
Documentary	3.00 (2.05%)	3.00 (5.66%)	0.00 (0.00%)	
Medical scene	26.00 (17.81%)	6.00 (11.32%)	20.00 (21.51%)	
Other	2.00 (1.37%)	2.00 (3.77%)	0.00 (0.00%)	
Monologue	79.00 (54.11%)	12.00 (22.64%)	67.00 (72.04%)	
PowerPoint	25.00 (17.12%)	24.00 (45.28%)	1.00 (1.08%)	
Q&A format	5.00 (3.42%)	2.00 (3.77%)	3.00 (3.23%)	
Video content (*N* = 282)				<0.001
Epidemiology	15 (5.14%)	11 (8.53%)	4 (2.45%)	
Etiology	18 (6.16%)	8 (6.20%)	10 (6.13%)	
Symptoms	124 (42.47%)	45 (34.88%)	79 (48.47%)	
Diagnosis	38 (13.01%)	19 (14.73%)	19 (11.66%)	
Treatment	56 (19.18%)	29 (22.48%)	27 (16.56%)	
Prevention	8 (2.74%)	4 (3.10%)	4 (2.45%)	
Prognosis	15 (5.14%)	1 (0.78%)	14 (8.59%)	
Follow-up	14 (4.79%)	8 (6.20%)	6 (3.68%)	
Nursing	4 (1.37%)	4 (3.10%)	0 (0.00%)	

### 3.3 Video quality across different video sources, formats, and content types

**Figure 2:**
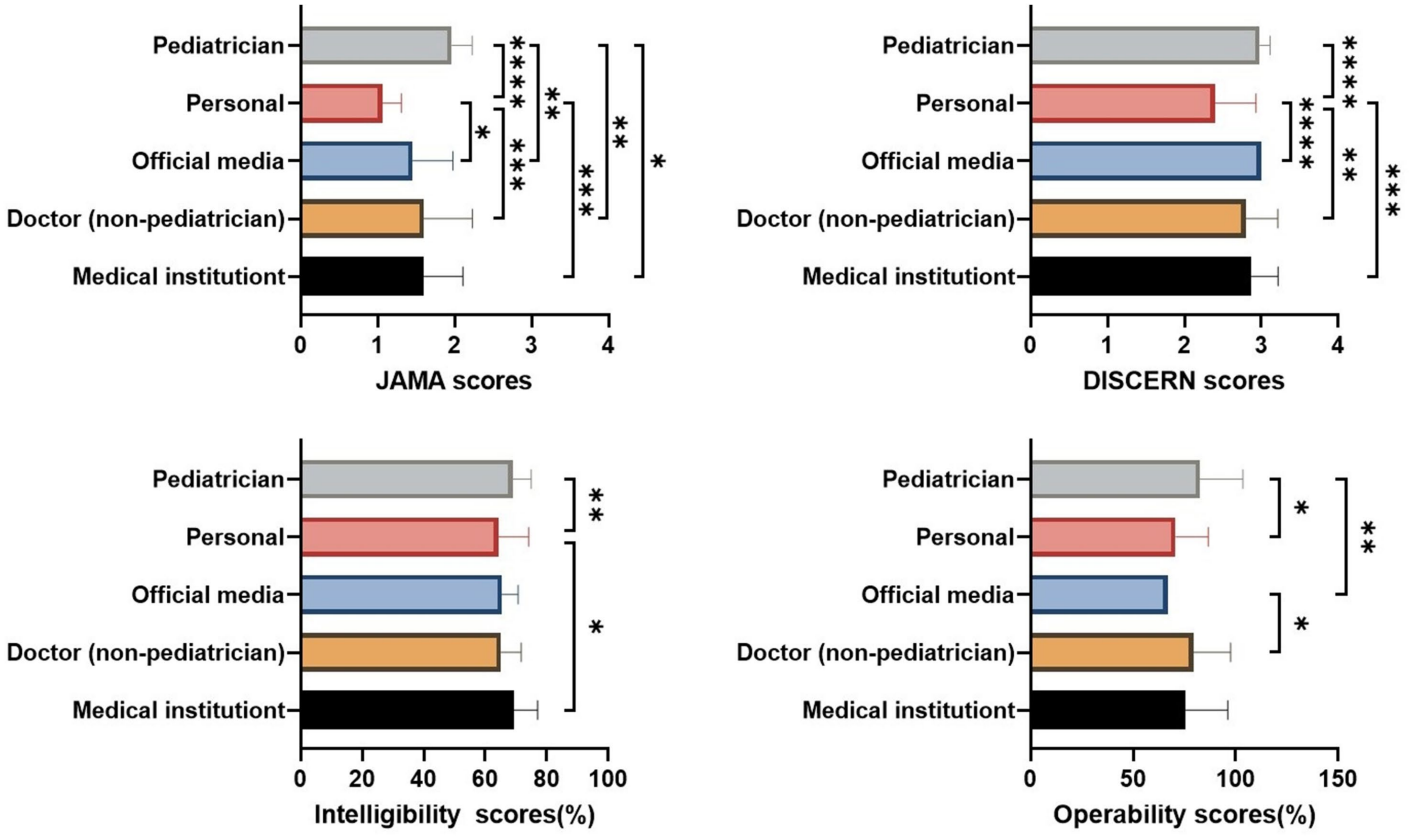
Quality of videos across different sources. *p < 0.05, **p < 0.005, ***p < 0.0005, ****p < 0.0001.

Overall data showed that the JAMA score for videos uploaded by pediatricians was significantly higher than those uploaded by personal users, official media, non-pediatrician doctors, and medical institutions (*p* < 0.0001, *p* < 0.005, *p* < 0.005, *p* < 0.05, respectively). Additionally, pediatricians’ videos had significantly higher modified DISCERN scores, intelligibility, and operability than those uploaded by personal users (*p* < 0.0001, *p* < 0.005, *p* < 0.05, respectively). Videos uploaded by personal users had significantly lower JAMA and modified DISCERN scores than those uploaded by official media (*p* < 0.05 and *p* < 0.0001, respectively) and non-pediatrician doctors (*p* < 0.0005 and *p* < 0.005, respectively). Furthermore, the JAMA score, modified DISCERN score, and intelligibility of videos uploaded by medical institutions were significantly higher than those uploaded by personal users (*p* < 0.0005, *p* < 0.0005, *p* < 0.05, respectively). Interestingly, the operability of videos uploaded by official media was lower than that of videos uploaded by pediatricians (*p* < 0.005) and non-pediatrician doctors (*p* < 0.05) (Figure 2).

The patterns observed on TikTok were generally consistent with the overall results. Notably, medical institutions demonstrated higher JAMA scores on TikTok but lower scores on Bilibili. In addition, personal users on Bilibili did not differ significantly from other uploader groups in terms of DISCERN scores, intelligibility, or operability (Supplementary Figures S1, S2).

**Figure 3:**
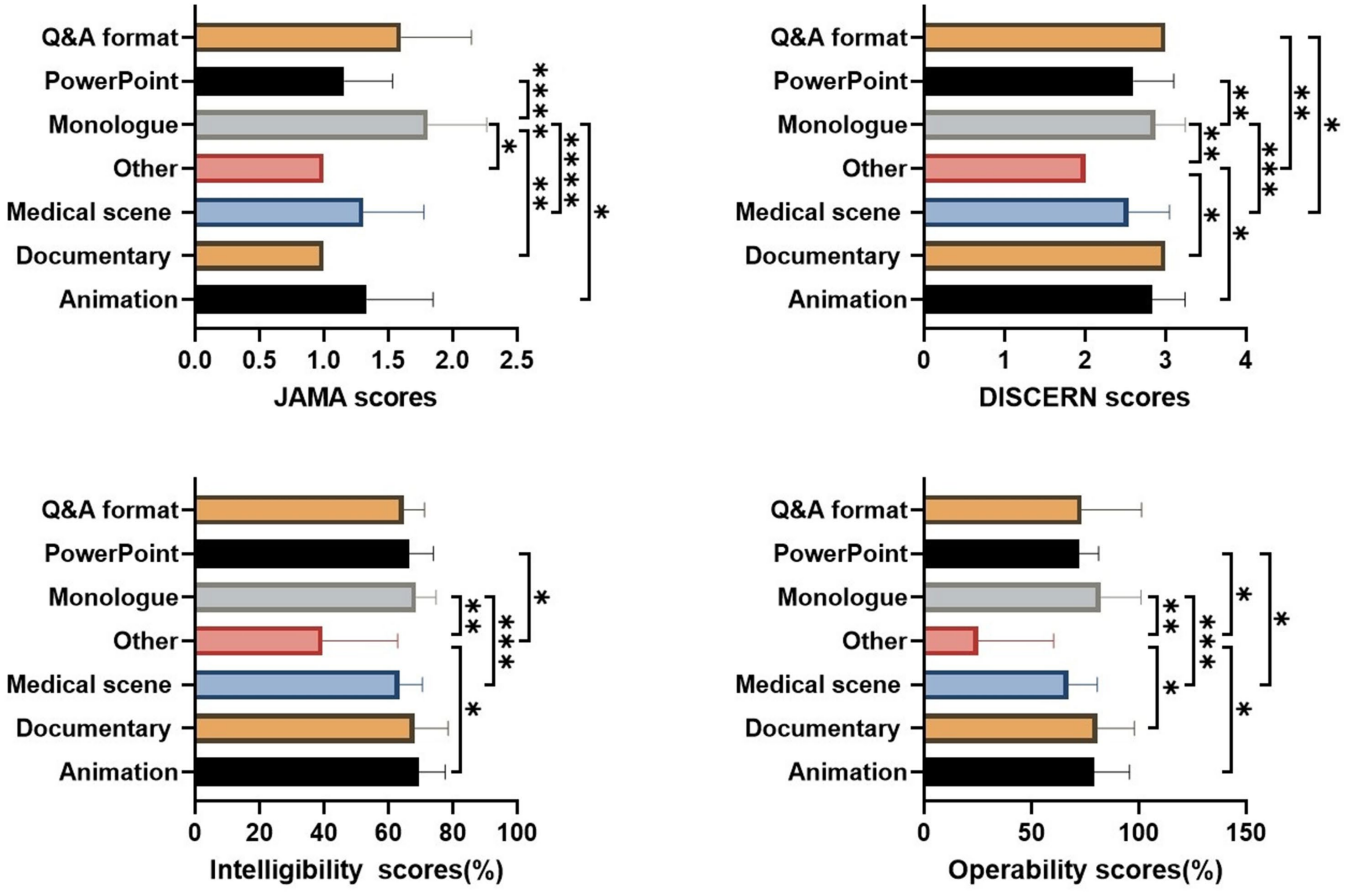
Quality of videos across different presentation formats. *p < 0.05, **p < 0.005, ***p < 0.0005, ****p < 0.0001.

For different video formats, the modified DISCERN score for Q&A format videos was significantly higher than that for other formats and medical scenes (*p* < 0.005 and *p* < 0.05, respectively). Videos in PowerPoint format had higher JAMA and modified DISCERN scores than those in Monologue format (*p* < 0.0001 and *p* < 0.005, respectively). At the same time, Monologue format videos had significantly higher JAMA scores, modified DISCERN scores, intelligibility, and operability than those in other formats (*p* < 0.05, *p* < 0.005, *p* < 0.005, *p* < 0.005, respectively) and medical scenes (*p* < 0.0001, *p* < 0.0005, *p* < 0.0005, *p* < 0.0005, respectively). Furthermore, Monologue format videos had higher JAMA scores than documentary and animation formats (*p* < 0.005 and *p* < 0.05, respectively). Additionally, documentary and animation formats outperformed other formats in modified DISCERN scores and operability (*p* < 0.05). PowerPoint videos were more understandable than other types (*p* < 0.05), and more operable compared to both other and medical scene formats (*p* < 0.05) (Figure 3).

Given that other and documentary formats videos were not present on TikTok, the differences in video format between the two platforms were more pronounced, although the groups showing significant variation were generally consistent with the overall distribution. On TikTok, videos in the medical scenes format scored significantly lower than monologue videos across all four dimensions. In addition, animation videos on TikTok demonstrated significantly higher operability compared with the Q&A, monologue, and medical scenes formats. On Bilibili, monologue videos exhibited relatively higher JAMA scores, whereas videos categorized as other showed significantly lower DISCERN scores, intelligibility, and operability compared with documentary, monologue, PowerPoint, and medical scene videos (Supplementary Figures S3, S4).

**Figure 4:**
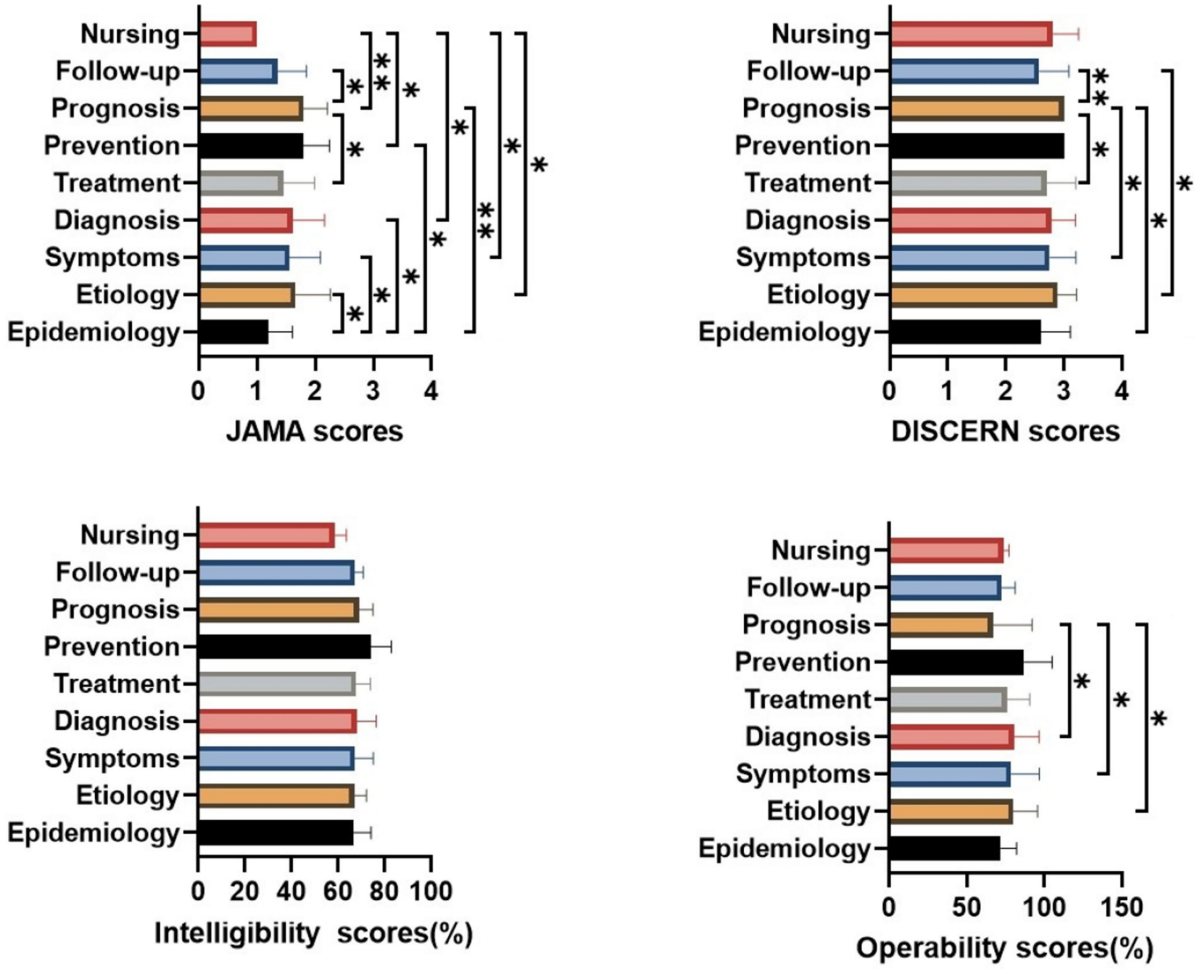
Quality of videos across different content types. *p < 0.05, **p < 0.005, ***p < 0.0005, ****p < 0.0001.

For videos with different content types, videos related to nursing had lower JAMA scores than those related to prognosis, prevention, diagnosis, symptoms, and etiology (*p* < 0.005, *p* < 0.05, *p* < 0.05, *p* < 0.05, *p* < 0.05, respectively). Compared to other content types, epidemiology-related videos had significantly lower JAMA scores than etiology, symptoms, diagnosis, prevention, and prognosis (*p* < 0.05, *p* < 0.05, *p* < 0.05, *p* < 0.05, *p* < 0.005, respectively). Notably, prognosis-related videos received higher JAMA scores (*p* < 0.05) and modified DISCERN scores (*p* < 0.005, *p* < 0.05) compared to follow-up and treatment videos. Moreover, prognosis-related videos had higher modified DISCERN scores than those related to symptoms and epidemiology (*p* < 0.05). However, prognosis-related videos had lower operability compared to diagnosis, symptoms, and etiology (*p* < 0.05). No significant differences were observed in intelligibility across different content types (Figure 4).

Although the pooled analyses showed differences in JAMA and DISCERN scores across video content types, platform-stratified analyses revealed that such differences were limited on TikTok, where only JAMA scores varied by content type—specifically, videos related to symptoms scored significantly lower than those focusing on etiology or diagnosis. Interestingly, although no significant differences in intelligibility were observed across content types in the overall dataset, TikTok and Bilibili demonstrated distinct patterns. On TikTok, diagnosis-related videos were more intelligible than those focusing on epidemiology or symptoms. In contrast, on Bilibili, prevention-related videos showed significantly higher intelligibility compared with nursing, etiology, and diagnosis videos. Moreover, nursing-related videos were less intelligible than those covering epidemiology, symptoms, and treatment, and treatment-related videos demonstrated significantly higher intelligibility than diagnosis videos. With respect to operability, the patterns observed on TikTok were consistent with the pooled analysis, whereas findings from Bilibili indicated that prevention videos had higher operability compared with those focused on prognosis (Supplementary Figures S5, S6).

### 3.4 Popularity across different video sources, formats, and content types

We analyzed the popularity of videos across different sources, presentation formats, and content types. Videos uploaded by pediatricians received significantly more likes and comments (*p* = 0.005 and *p* = 0.03, respectively). Meanwhile, videos from official media sources gained more shares, while those uploaded by personal users tended to have longer durations. Audience preferences varied across different video formats. Monologue-style videos were more likely to receive likes and shares (*p* < 0.001), whereas medical scene videos attracted more comments (*p* < 0.001). PowerPoint-based videos generally had longer durations. Regarding video content, videos addressing etiology were more likely to receive likes, comments, and saves (*p* < 0.001, *p* = 0.002, and *p* = 0.002, respectively), while those focusing on prevention received more shares. Notably, videos related to nursing had the longest duration (Table 3).

**Table 3:** Popularity of videos across different video sources, formats, and content types.

Variable	Likes, median (p25–p75)	Comments, median (p25–p75)	Shares, median (p25–p75)	Saves, median (p25–p75)	Time, median (p25– p75)
Video source (*N* = 146)					
Medical institution (*n* = 15)	33.00 (17.00–417.00)	2.00 (0.00–61.00)	89.00 (3.00–195.00)	48.00 (6.00–104.00)	111.00 (66.00–218.00)
Doctor (non-pediatrician) (*n* = 15)	93.00 (30.00–1,956.00)	5.00 (2.00–71.00)	25.00 (8.00–517.00)	85.00 (20.00–1,290.00)	150.00 (78.00–240.00)
Official media (*n* = 11)	200.00 (47.00–26,367.00)	10.00 (1.00–2,051.00)	246.00 (28.00–18,024.00)	27.00 (5.00–11,785.00)	27.00 (17.00–93.00)
Personal (*n* = 48)	47.50 (10.00–398.00)	9.50 (1.00–70.50)	28.00 (8.50–89.00)	31.00 (10.50–112.00)	269.50 (91.50–747.00)
Pediatrician (*n* = 57)	300.00 (55.00–1,716.00)	37.00 (6.00–224.00)	221.00 (31.00–962.00)	106.00 (19.00–445.00)	87.00 (50.00–121.00)
*p*-value	0.005	0.03	<0.001	0.076	<0.001
Video presentation format (*N* = 146)					
Animation (*n* = 6)	98.50 (33.00–115.00)	4.00 (0.00–10.00)	103.50 (89.00–115.00)	55.00 (25.00–69.00)	329.50 (127.00–1,231.00)
Documentary (*n* = 3)	13.00 (7.00–3,505.00)	1.00 (0.00–704.00)	29.00 (15.00–523.00)	16.00 (7.00–335.00)	202.00 (51.00–3,721.00)
Medical scene (*n* = 26)	234.00 (47.00–1,310.00)	65.50 (3.00–364.00)	79.00 (24.00–839.00)	45.00 (9.00–629.00)	47.50 (27.00–162.00)
Other (*n* = 2)	304.00 (12.00–596.00)	10.00 (0.00–20.00)	9.00 (9.00–9.00)	40.50 (28.00–53.00)	282.00 (256.00–308.00)
Monologue (*n* = 79)	314.00 (51.00–1,956.00)	42.00 (5.00–261.00)	195.00 (28.00–962.00)	106.00 (17.00–445.00)	93.00 (56.00–150.00)
PowerPoint (*n* = 25)	15.00 (4.00–46.00)	1.00 (0.00–2.00)	15.00 (4.00–24.00)	29.00 (8.00–51.00)	487.00 (177.00–1,785.00)
Q&A format (*n* = 5)	55.00 (3.00–513.00)	5.00 (1.00–38.00)	46.00 (2.00–80.00)	22.00 (3.00–85.00)	49.00 (46.00–246.00)
*p*-value	<0.001	<0.001	<0.001	0.184	<0.001
Video content (*N* = 282)					
Diagnosis (*n* = 36)	71.50 (28.00–452.50)	7.50 (1.00–66.00)	32.50 (14.50–250.50)	63.50 (32.50–165.00)	152.50 (93.50–624.50)
Epidemiology (*n* = 15)	46.00 (12.00–506.00)	5.00 (0.00–41.00)	31.00 (15.00–246.00)	47.00 (26.00–335.00)	299.00 (73.00–2,080.00)
Etiology (*n* = 18)	349.00 (44.00–545.00)	31.50 (10.00–89.00)	103.50 (26.00–536.00)	131.00 (38.00–392.00)	173.50 (93.00–1,231.00)
Follow-up (*n* = 14)	23.50 (6.00–55.00)	2.00 (0.00–6.00)	17.00 (8.00–28.00)	12.50 (5.00–28.00)	437.50 (121.00–2,565.00)
Nursing (*n* = 5)	6.00 (2.00–8.00)	0.00 (0.00–0.00)	8.00 (4.00–13.00)	16.00 (6.00–18.00)	561.00 (207.00–646.00)
Prevention (*n* = 5)	109.00 (24.00–315.00)	3.00 (2.00–79.00)	144.00 (29.00–210.00)	69.00 (16.00–92.00)	162.00 (103.00–202.00)
Prognosis (*n* = 15)	128.00 (40.00–493.00)	18.00 (3.00–172.00)	122.00 (28.00–447.00)	47.00 (12.00–112.00)	56.00 (46.00–121.00)
Symptoms (*n* = 121)	130.00 (31.00–657.00)	20.00 (2.00–216.00)	80.00 (20.00–523.00)	53.00 (16.00–335.00)	102.00 (56.00–256.00)
Treatment (*n* = 53)	44.00 (13.00–128.00)	3.00 (1.00–28.00)	28.00 (13.00–76.00)	28.00 (12.00–61.00)	186.00 (93.00–547.00)
*p*-value	<0.001	0.002	0.002	0.002	<0.001

Platform-stratified analyses revealed notable heterogeneity. On Bilibili, videos uploaded by doctors (non-pediatricians) received more comments, and animation videos generated more shares. On TikTok, videos related to epidemiology achieved higher numbers of likes, shares, and saves (Supplementary Tables S4, S5).

### 3.5 Variation in video formats across different sources

There were significant differences in video presentation formats among different uploader types. Monologue was the most commonly used format by medical institutions, non-pediatrician doctors, and pediatricians (6/15, 40.00%; 11/15, 73.33%; and 50/57, 87.72%, respectively). In contrast, the medical scene format was predominantly favored by official media sources (9/11, 81.82%). Personal users most frequently opted for the PowerPoint format (15/48, 31.25%). Although the video content varied significantly across different uploader types, all groups primarily focused on symptoms (Table 4).

**Table 4:** Variation in video formats across different sources.

Variable	Medical institution	Doctor (non-pediatrician)	Official media	Personal	Pediatrician	*p*-value
Video presentation format (*N* = 146)						<0.001
Animation	3.00 (20.00%)	0.00 (0.00%)	0.00 (0.00%)	3.00 (6.25%)	0.00 (0.00%)	
Documentary	0.00 (0.00%)	0.00 (0.00%)	0.00 (0.00%)	3.00 (6.25%)	0.00 (0.00%)	
Medical scene	1.00 (6.67%)	0.00 (0.00%)	9.00 (81.82%)	13.00 (27.08%)	3.00 (5.26%)	
Other	0.00 (0.00%)	0.00 (0.00%)	0.00 (0.00%)	2.00 (4.17%)	0.00 (0.00%)	
Monologue	6.00 (40.00%)	11.00 (73.33%)	2.00 (18.18%)	10.00 (20.83%)	50.00 (87.72%)	
PowerPoint	4.00 (26.67%)	3.00 (20.00%)	0.00 (0.00%)	15.00 (31.25%)	3.00 (5.26%)	
Q&A format	1.00 (6.67%)	1.00 (6.67%)	0.00 (0.00%)	2.00 (4.17%)	1.00 (1.75%)	

### 3.6 Correlation analysis

**Figure 5:**
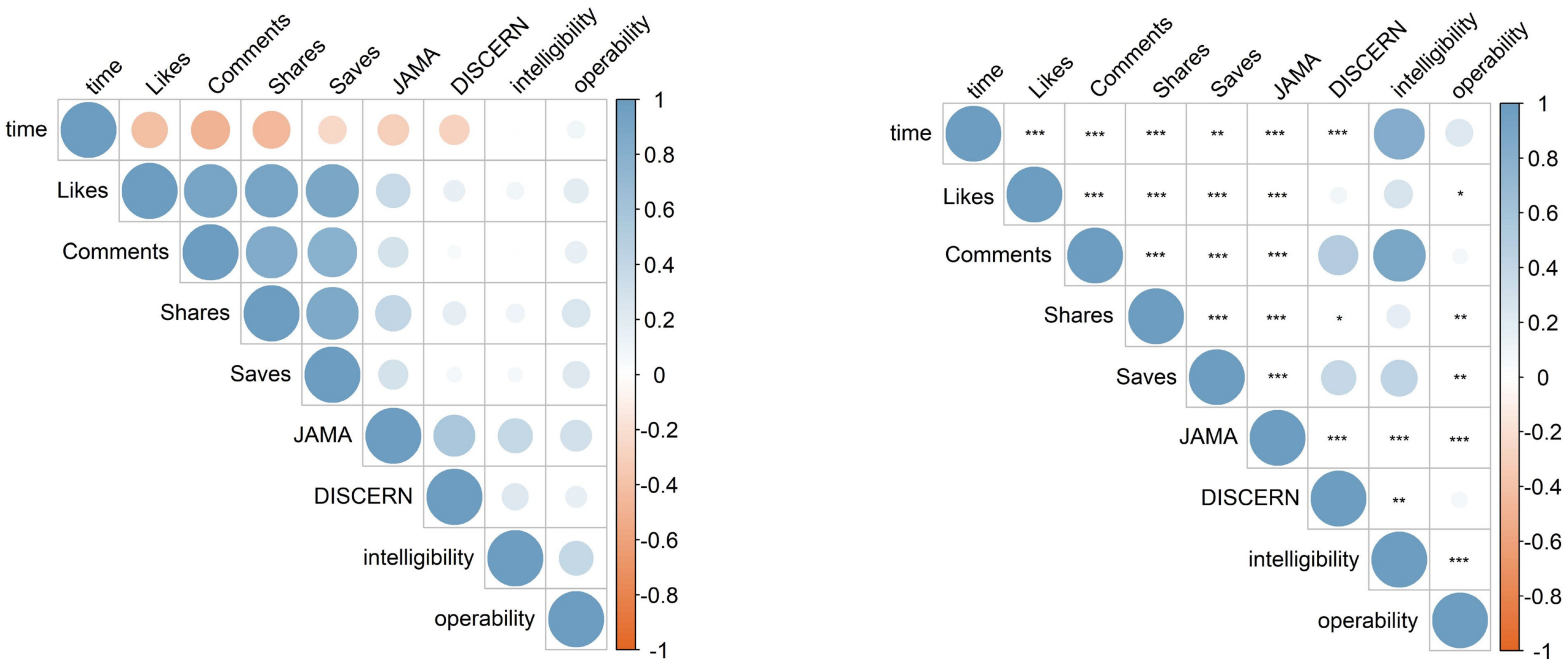
Correlation analysis between video variables and video quality.

Spearman correlation analysis revealed associations between video popularity metrics and quality scores. Likes, comments, shares, and saves were all positively correlated with one another (all *p* < 0.001). Video duration showed a negative correlation with likes, comments, shares, and saves (all *p* < 0.01). Higher JAMA scores were associated with greater numbers of likes, comments, shares, and saves (all *p* < 0.001). Videos with higher modified DISCERN scores were only associated with more shares (*p* < 0.05). Notably, longer videos were negatively correlated with both JAMA and modified DISCERN scores (all *p* < 0.001). Intelligibility showed no significant association with likes, comments, shares, or saves (all *p* > 0.05), whereas higher operability was associated with more likes, shares, and saves (all *p* < 0.05). Video duration was not significantly correlated with either intelligibility or operability (Figure 5).

Although video duration on TikTok remained negatively correlated with likes, comments, shares, saves, and the modified DISCERN score, this pattern was not observed on Bilibili. In addition, JAMA scores showed no independent associations with likes, comments, shares, or saves on either platform. Notably, on TikTok, higher intelligibility was associated with longer video duration and fewer comments. While no association between video duration and operability was observed in the pooled analysis, platform-specific analyses on both platforms indicated that higher operability was associated with longer video duration (Supplementary Figure S8).

### 3.7 Qualitative characterization of misinformation themes

Overall, 64.58% (31/48) of videos uploaded by individual users were identified as containing misinformation. Within these videos, five major misinformation themes were identified: (1) oversimplification of clinical symptoms (*n* = 8); (2) incorrect etiological claims (*n* = 4); (3) promotion of non–evidence-based home treatments (*n* = 6); (4) misunderstanding of diagnostic criteria (*n* = 10); and (5) misleading statements regarding immunoglobulin therapy (*n* = 6). Because a single video could encompass multiple themes, the total number of themes exceeded the number of videos. Details of the themes and their descriptions are presented in Table 5.

**Table 5:** Classification of misinformation themes among individual uploaders.

Theme	Description	Representative examples from videos (Paraphrased)
Oversimplification of clinical symptoms	Kawasaki disease described as a mild viral illness or simple fever	“Just a normal viral fever; no need to worry about complications”
Incorrect etiological claims	Unverified causes or triggers falsely linked to the disease	“Kawasaki disease is caused by eating too much seafood”
Promotion of non–evidence-based home treatments	Alternative therapies suggested as replacements for medical care	“You can cure it with herbal water at home; no need to go to hospital”
Misinterpretation of diagnostic criteria	Diagnostic steps oversimplified or incorrectly explained	“If your child has rash and fever, it’s definitely Kawasaki disease”
Misleading statements regarding immunoglobulin therapy	Risks exaggerated or treatment necessity denied	“IVIG is extremely dangerous—many children do not need it at all”

## 4 Discussion

Kawasaki disease is an acute vasculitis that primarily affects children. If not promptly recognized and treated, it can lead to serious coronary artery complications, making public awareness of the disease critically important. With the rapid development of the internet, social media platforms have become major sources for health information, especially among younger populations (22). However, despite the convenience and accessibility of these platforms, the reliability and quality of their content remain areas of concern.

In this study, we conducted a comprehensive analysis of videos related to Kawasaki disease on Bilibili and TikTok, evaluating their content, reliability, and overall quality. Our findings showed that videos on TikTok were significantly more popular, receiving higher numbers of likes, comments, shares, and saves compared to those on Bilibili. Nevertheless, the overall quality of videos on both platforms was suboptimal, particularly those on Bilibili. Pediatricians and individual users were the two main sources of video content, with most videos presented in a monologue format and primarily focusing on symptoms. Videos uploaded by pediatricians and official media were generally more favored by users, with those created by pediatricians demonstrating the highest quality. In terms of presentation formats and content types, monologue-style videos and those focusing on disease etiology were both more popular and of higher quality. These findings suggest that although social media plays an increasingly vital role in public health education, there remain notable differences in content presentation, quality, reliability, and popularity across different platforms and content creators. These disparities warrant further investigation and targeted interventions.

### 4.1 Factors associated with video quality

Our study highlights concerns regarding the quality of videos related to Kawasaki disease, aligning with findings from other studies on short video content. For instance, Yeung et al. analyzed short videos focusing on ADHD and were surprised to find that approximately half of the TikTok videos were misleading (14). Similarly, Sturm et al.’s study on the content quality of pediatric nephrology short videos warned educators about the lack of reliability in medical information provided on TikTok (23). In our research, we found that the professional background of video creators significantly influenced video quality. Compared to other types of creators, pediatricians’ videos were of markedly higher quality, offering more useful information with fewer misleading elements. This may be attributed to pediatricians’ solid expertise and their precise understanding of the health education needs of the target audience (24). Moreover, we found that videos uploaded by pediatricians scored higher on the JAMA criteria and were more operationally feasible than those from official media, which contradicts a study on the quality of colorectal polyp videos (7). This phenomenon suggests that pediatricians are producing higher-quality videos about Kawasaki disease on both platforms.

The overall quality of videos uploaded by individual users was relatively low. This phenomenon may stem from the fact that most individual creators lack medical training, making their content more prone to bias in terms of information selection, organization, and expression (25). We observed that most individual uploaders identified themselves as parents of children. As a result, their videos often emphasized emotional expression and personal experiences rather than informational accuracy and educational value.

Furthermore, our qualitative review of individual videos revealed that a significant number contained misleading information, including several recurring misinformation themes. First, the clinical manifestations of Kawasaki disease were frequently oversimplified, with some creators depicting it as a common viral infection or a self-limiting febrile illness. However, such simplification may impede the timely recognition of warning signs, particularly those related to coronary artery involvement (3). Second, unverified or incorrect etiological explanations were common, including attributing Kawasaki disease to specific foods, seasonal factors, or parental behaviors. Third, some videos promoted non–evidence-based home remedies—such as herbal preparations, dietary interventions, or massage therapy—as alternatives to standard medical care. Individual users often lack awareness of established guidelines and the nature of the disease, leading to incorrect attributions and the promotion of unverified therapies. This may distort caregivers’ perceptions of risk factors, reinforce false beliefs about preventability (26), and impede effective early management (27). Fourth, misunderstandings of diagnostic criteria were frequently observed. This suggests that many creators lack adequate knowledge of the clinical thresholds necessary for identifying Kawasaki disease. Such misinformation may inadvertently encourage caregivers to rely on incomplete symptom recognition, thereby delaying appropriate evaluation in clinical settings (28). Finally, some videos included inaccurate statements regarding intravenous immunoglobulin (IVIG). This is particularly concerning. As IVIG remains the cornerstone of Kawasaki disease management (29), misinformation that exaggerates its risks or minimizes its necessity may undermine caregivers’ acceptance of evidence-based treatment. These misinformation patterns may significantly influence caregivers’ decision-making, potentially provoke health-related anxiety, and increase unnecessary healthcare utilization (30). Notably, TikTok’s unique algorithm has been shown to promote similar content based on users’ watch history, which may further exacerbate the spread of misleading videos (31). Fortunately, our study found that videos by individual users were generally less popular—a finding that contrasts with research on ADHD-related content (14). However, a concerning phenomenon emerged: some individual users appeared to misrepresent their identities, posing as unverified healthcare professionals. This may be a strategy to boost viewership and credibility, but it risks compromising the quality of online health information and undermining the professional image of healthcare providers (32). Therefore, when seeking health information on video-sharing platforms, users should pay close attention to the uploader’s identity verification.

Videos related to nursing and epidemiology generally received lower JAMA scores. In contrast, videos focusing on prognosis, etiology, diagnosis, and prevention achieved higher scores in terms of information quality and reliability. This discrepancy may be attributed to both platform content preferences and the structural criteria of the JAMA scoring system. Firstly, as video platforms, TikTok and Bilibili emphasizes content visibility and dissemination efficiency (33). When searching for information on Kawasaki disease, the public tends to focus on key questions such as “Could my child have this disease?,” “What causes it?,” and “Will there be long-term consequences?” These concerns drive content creators to devote more attention to such topics, which are more likely to receive algorithmic promotion and thus be optimized to higher quality standards. In contrast, content on nursing procedures and epidemiological features is often presented as supplementary background information, lacking systematic explanation, which negatively impacts overall content quality scores. Secondly, compared to nursing-related videos, those discussing etiology, diagnostic processes, and treatment outcomes—topics considered core medical knowledge—tend to be more structured, authoritative, and include more source citations, making them more aligned with the JAMA assessment criteria (17). Consequently, even if nursing and epidemiology-related videos possess practical value, their lower degree of structural organization may place them at a disadvantage in standardized quality evaluations.

Moreover, across all video categories—including those related to nursing and epidemiology—the JAMA scores were generally lower compared to the modified DISCERN scores. This discrepancy primarily reflects the differing emphases of the two evaluation tools. As mentioned above, the JAMA scoring system focuses more on the formal attributes of information source transparency and accountability, such as authorship disclosure, source citation, date of last update, and the presence of disclaimers (17). However, such elements are rarely included in videos related to Kawasaki disease, resulting in lower scores under the JAMA framework. In contrast, the DISCERN tool places greater emphasis on the substance and practical utility of the content, including completeness, relevance, and its usefulness in supporting informed health decision-making by the public (18). For example, although videos on topics such as nursing and prognosis may lack adequate referencing or disclosure, they often address real-world concerns of viewers—such as home care practices, prognostic expectations, and the balance of risks and benefits. These videos tend to be highly practical, which contributes to better performance on DISCERN criteria.

Notably, although prognosis-related videos demonstrated superior information quality, they performed relatively poorly in terms of operability compared to content focusing on symptoms, diagnosis, and etiology. This may be attributed to the self-limiting nature of Kawasaki disease itself (34). In addition, prognosis-related content often emphasizes the disease trajectory, long-term outcomes, and probabilistic assessments, rather than offering concrete and actionable recommendations (35).

### 4.2 Factors associated with popularity

Studies have shown that the number of likes, comments, saves, and shares can partially reflect a video’s popularity (36). In our analysis, some Kawasaki disease-related videos received as many as 144,650 likes and 139,899 shares, with comments and shares in some cases exceeding 13,000 and 17,000, respectively, indicating a high level of public interest in the topic. Further analysis of the relationship between popularity and the video’s source, format, and content revealed that the professionalism of the information source may influence user engagement. Although pediatricians and individual users contributed a similar number of videos, those uploaded by pediatricians received significantly more likes and comments, possibly because users are more inclined to trust content provided by professionals (37). Official media were more likely to trigger sharing behaviors, which may reflect their inherent advantages in content dissemination. In contrast, although videos uploaded by individual users tended to be longer, they did not result in higher engagement, suggesting that video length may not compensate for the lack of credibility due to insufficient expertise.

The format in which a video is presented also significantly influences its popularity. Monologue videos, not only being the most common format, also exhibited relatively high quality. Moreover, they were more likely to receive likes and shares. This may be attributed to the first-person narrative style commonly used in monologues (14), which fosters a more direct and intimate form of communication and facilitates the delivery of health information (7). This effect is particularly pronounced when the presenter is a pediatrician, as monologue videos can convey professionalism while simultaneously bridging the psychological gap with viewers. As a result, this format is also favored by medical institutions and non-pediatrician doctors. Medical scene videos, on the other hand, were associated with a significantly higher number of comments, suggesting that they may evoke more emotional resonance or questions among viewers, thereby stimulating discussion. Interestingly, although the PowerPoint format did not differ from the medical scene format in popularity metrics, it demonstrated stronger operability. This could be due to its clear structure and logical organization, which are well-suited for conveying treatment plans and scientific presentations (38). However, it is worth noting that PowerPoint-based videos tend to be longer in duration, potentially demanding more sustained attention from viewers.

Although videos related to symptoms were the most frequently uploaded, content addressing etiology was significantly associated with a higher number of likes, comments, and saves, indicating a strong user interest in the underlying mechanisms of the disease. However, as the etiology of Kawasaki disease remains unclear (39), such videos should be interpreted with caution. Videos focused on prevention received more shares. In clinical practice, prevention of coronary artery aneurysms is a central goal in the treatment of Kawasaki disease (40), as these aneurysms represent the leading cause of both short-term and long-term mortality in affected children (41). Therefore, content related to preventive measures has high practical value and public health relevance, which may prompt users to actively disseminate such information.

A paradoxical finding is that although prognosis-related videos demonstrated high quality, they were less popular than those related to etiology and prevention. Indeed, prognosis is rarely the subject of systematic and sustained multidisciplinary research. Nonetheless, the prognostic aspects of Kawasaki disease should not be overlooked. Several studies have revealed the potential risks of coronary artery occlusion (42), giant coronary aneurysms (43), and silent myocardial infarction (44), with some reporting an increased risk of allergic diseases in affected patients (45). Therefore, videos discussing prognosis can play a crucial role in informing caregivers about possible long-term outcomes and guiding rehabilitation efforts.

### 4.3 Correlation analysis

Likes, comments, shares, and saves were positively correlated with each other and also showed significant positive correlations with video quality. In other words, the number of likes and comments tended to increase in parallel with the number of shares and saves. These findings indicate that reliability and operability are critical factors influencing popularity. The typical user engagement path for high-quality videos—like-comment-save-share—may significantly optimize information dissemination. However, intelligibility was not associated with video popularity, suggesting that this dimension still requires improvement in short-form health communication, especially as a potential entry point for translating professional content to lay audiences. In our study, the longest video duration reached 3,721 s. Further analysis revealed that video duration was negatively correlated with the number of likes, comments, shares, saves, as well as JAMA and modified DISCERN scores. This finding aligns with a previous YouTube-based study assessing video quality for sinusitis (46), and underscores the current user preference for concise and focused content (47). Therefore, balancing video length with audience retention may be a key strategy to enhance both popularity and quality of health education videos.

### 4.4 Platform-specific differences

Our platform-stratified analyses revealed meaningful heterogeneity in video quality, content characteristics, and engagement patterns between TikTok and Bilibili, underscoring the importance of accounting for platform-specific contexts when interpreting short-form health information.

Although the overall patterns were largely consistent across platforms, notable discrepancies emerged for certain uploader groups. In particular, medical institutions achieved higher JAMA scores on TikTok but lower scores on Bilibili. This divergence may reflect differences in platform content ecology and audience expectations. TikTok’s algorithm favors concise, structured, and visually engaging content (48), which may allow institutional accounts to present guideline-aligned information in a standardized format. In contrast, Bilibili users often expect in-depth explanations and narrative coherence, potentially placing institutional content at a relative disadvantage when it lacks sufficient depth or interactivity.

Differences in video format were more pronounced across platforms, partly because certain formats (e.g., documentary and “other” formats) were absent on TikTok. Despite this structural divergence, the formats showing lower quality scores were broadly consistent with the pooled analysis. On TikTok, animation-based videos demonstrated higher operability on TikTok, likely because animated formats facilitate stepwise presentation and simplify procedural guidance within short time constraints. On Bilibili, monologue-style videos were associated with higher JAMA scores, supporting the notion that this platform favors structured, presenter-centered explanations.

Although pooled analyses suggested differences in JAMA and DISCERN scores across content types, platform-stratified analyses revealed that such differences were primarily driven by TikTok, where symptom-focused videos had significantly lower JAMA scores than those addressing etiology or diagnosis. This finding suggests that symptom-oriented content may be more prone to oversimplification or omission of sourcing when tailored for rapid consumption.

Interestingly, intelligibility patterns diverged substantially across platforms. While no overall association was observed in pooled data, TikTok users found diagnosis-related videos more intelligible than those focused on epidemiology or symptoms, possibly reflecting audience preference for concrete, decision-oriented information. In contrast, on Bilibili, prevention-related videos were the most intelligible, surpassing nursing, etiology, and diagnosis content. This may reflect Bilibili users’ greater tolerance for longer, explanatory narratives and their interest in proactive disease management. Furthermore, nursing-related videos were consistently less intelligible than epidemiology, symptom, and treatment videos, suggesting that caregiving guidance may require more structured presentation to be effectively understood.

With respect to operability, TikTok exhibited patterns similar to the pooled analysis, whereas Bilibili uniquely showed higher operability for prevention-related videos compared with prognosis-focused content. This suggests that actionable preventive guidance may be more readily translated into practical steps by Bilibili audiences.

Platform-specific analyses also highlighted distinct engagement mechanisms. On TikTok, video duration remained negatively correlated with likes, comments, shares, saves, and modified DISCERN scores, consistent with the platform’s preference for brevity and rapid content turnover (49). This relationship was absent on Bilibili, where longer videos are more culturally accepted (50). Importantly, JAMA scores were not independently associated with engagement metrics on either platform, reinforcing the notion that popularity does not reliably reflect informational quality. Notably, on TikTok, higher intelligibility was associated with longer video duration and fewer comments, suggesting that clearer videos may reduce the need for viewer clarification or debate. Although pooled analyses did not reveal an association between duration and operability, both platforms independently demonstrated that longer videos were associated with higher operability. This finding highlights the presence of a classic Simpson’s paradox between platform-specific and pooled analyses (51) and further suggests that providing actionable medical guidance may inherently require additional time, regardless of the platform.

### 4.5 Our recommendations

Although the dissemination of health-related information on short video platforms is becoming increasingly popular, it still faces numerous challenges—particularly in balancing scientific accuracy and professionalism with effectively attracting and maintaining audience engagement. The involvement of specialist physicians is crucial for enhancing the impact of health education content. Currently, despite the high level of attention paid to Kawasaki disease on these platforms, the participation of pediatricians in such content remains significantly low, revealing a mismatch between high demand and low supply. Therefore, we call on more pediatric specialists to actively engage in the creation of high-quality short videos, aiming to achieve a synergy between professional accuracy and communication effectiveness.

At the same time, given the wide scope and complexity of information related to Kawasaki disease, establishing a unified and standardized content classification system would help users access the information they need more efficiently and enhance information retrieval. Considering that the overall quality of current videos still needs improvement, we recommend that in addition to relying on content creators’ own awareness of quality, platforms should implement measures such as regular training and professional content review mechanisms to guide creators in improving the scientific accuracy and reliability of their content. Moreover, when evaluating health-related short videos, it is important to recognize the differences in applicability and focus among various assessment tools. We suggest promoting the development of a comprehensive evaluation system adapted to diverse content types—one that emphasizes both formal standards and practical value.

To enhance the effectiveness of dissemination, we recommend fully leveraging the algorithmic advantages of platforms like TikTok to activate the four-dimensional engagement mechanism of “likes–comments–saves–shares.” Coupled with effective science communication strategies, this can significantly improve the visibility of video content and its reach among target audiences. Finally, our study highlights that controlling video length is crucial. Delivering high-quality, easy-to-understand information in a concise and appropriate format is key to the effective dissemination of health information. At the same time, over-simplification should be avoided to ensure that the scientific accuracy and integrity of the content are not compromised.

Platform-stratified analyses further emphasize that effective digital health communication cannot rely on a one-size-fits-all approach. Platform heterogeneity shapes not only content visibility but also how information is interpreted and translated into potential health behaviors. For public health practitioners, this underscores the need to adapt messaging strategies to platform-specific affordances, balancing brevity with clarity on TikTok while leveraging narrative depth and instructional detail on Bilibili. For researchers, our results highlight the importance of platform-stratified analyses to avoid overgeneralization and to ensure that public health recommendations are grounded in the realities of contemporary media consumption.

This study is the first cross-sectional analysis to systematically evaluate the quality and reliability of short videos related to Kawasaki disease using the JAMA Benchmark Criteria, the modified DISCERN tool, and the PEMAT instrument. However, several limitations should be acknowledged. First, the study only included the top 100 ranked videos from two Chinese short video platforms (TikTok and Bilibili), which may not fully represent the entire range of available content. Considering that most users tend to view only the first few pages of search results, our findings may better reflect the type of information most commonly accessed by the general public. Second, although inter-rater reliability was high between the two evaluators, video quality assessment inevitably involves some degree of subjectivity, which may introduce cognitive bias. Additionally, as all evaluators in this study were medical professionals, their deeper understanding of medical content may not accurately represent the perspectives of general viewers when assessing video intelligibility and operability. Future research involving laypersons as independent evaluators is warranted to ensure that the assessment outcomes more accurately reflect real-world viewer experience.

## 5 Conclusion

This study evaluated the quality and popularity of short videos related to Kawasaki disease on TikTok and Bilibili, providing several key insights into the dissemination of medical information about the disease. Most of the videos were uploaded by pediatricians and individual users, and typically conveyed symptom-related content in a monologue format. Although a limited number of videos created by pediatricians demonstrated higher quality and popularity, the overall reliability and intelligibility of the videos still require improvement. It is important to select appropriate evaluation tools based on the type and content of the videos. Notably, there was a significant positive correlation between video interactivity and quality, while video duration was negatively associated with both popularity and quality scores. These findings suggest that users should be cautious and selective when viewing Kawasaki disease-related content on short video platforms. Videos with shorter durations and higher interactivity may be a better choice. Platform heterogeneity further suggests that the effectiveness, interpretation, and potential behavioral impact of health information are context-dependent, shaped by platform-specific audience expectations, algorithmic mechanisms, and communication affordances. Balancing the depth and accuracy of medical information with audience receptivity remains a key challenge for content creators. Future research should aim to better understand the prevalence and nature of misinformation about Kawasaki disease on short video platforms.

## Data Availability

The original contributions presented in the study are included in the article/Supplementary material, further inquiries can be directed to the corresponding author/s.
