# Supplementary material for: Evaluating the quality and reliability of Kawasaki disease–related content on TikTok and Bilibili: a cross-sectional study
Source: Front Public Health. 2026 Jan 22;13:1664542. doi: 10.3389/fpubh.2025.1664542 (PMC12872735; doi:10.3389/fpubh.2025.1664542)
Supplement: Supplementary file 1 [file Supplementary_file_1.doc]

Multimedia Appendices

Table S1 Classification Criteria for Video Source, Content, and Presentation Format

| Category | Subcategory | Definition/Description |
| --- | --- | --- |
| Uploader Type | Pediatricians | Licensed physicians specializing in pediatrics. |
|  | Doctor (non-pediatrician) | Licensed medical professionals from other specialties. |
|  | Medical institution | Hospitals, clinics, or other formal healthcare organizations. |
|  | Personal | Individual users without verified medical background. |
|  | Official media | Government or mainstream media accounts with official certification. |
| Content Theme | Epidemiology | Information on disease incidence, prevalence, or demographic patterns. |
|  | Etiology | Explanation of possible causes or risk factors of Kawasaki disease. |
|  | Symptoms | Clinical manifestations and signs of the disease. |
|  | Diagnosis | Diagnostic criteria, laboratory tests, or imaging findings. |
|  | Treatment | Therapeutic approaches, including medication and other interventions. |
|  | Prevention | Measures to prevent the occurrence or complications of Kawasaki disease. |
|  | Prognosis | Expected disease outcome and long-term effects. |
|  | Follow-up | Information on post-treatment monitoring and check-ups. |
|  | Nursing | Nursing care, daily management, and patient support. |
| Presentation Format | Monologue | Single person speaking directly to the camera. |
|  | Q&A format | Question-and-answer style explanations, often simulated or scripted. |
|  | PowerPoint | Use of slide-based visuals to support verbal explanations. |
|  | Animation | Use of animated characters or illustrations to explain content. |
|  | Medical scene | Real-world hospital or clinic settings, including procedures or doctor-patient interactions. |
|  | Documentary | Narrative-driven format with interviews, footage, and storytelling. |
|  | Others | Content that does not fall into the above categories. |

Table S2 The Journal of the American Medical Association (JAMA) benchmark criteria

| Score* | Subcategory | Score component |
| --- | --- | --- |
| 1 score | Authorship | Author and contributor credentials and their affiliations should be provided. |
| 1 score | Attribution | Clearly lists all copyright information and states references and sources for content. |
| 1 score | Currency | Initial date of posted content and subsequent updates to content should be provided. |
| 1 score | Disclosure | Conflicts of interest, funding, sponsorship, advertising, support, and video ownership should be fully disclosed. |

*The criteria of each aspect were scored separately, and 1 point for each criterion with a total score of 4 points.

Table S3 modified DISCERN tool

| Score* | Reliability Score |
| --- | --- |
| 1 score | Is the video clear, concise, and understandable? |
| 1 score | Are reliable sources of information used? (i.e., publication cited, speaker is specialist) |
| 1 score | Is the information presented balanced and unbiased? |
| 1 score | Are additional sources of information listed for patient reference? |
| 1 score | Are areas of uncertainty/controversy mentioned? |

*The criteria of each aspect were scored separately, and 1 point is given for every Yes and 0 points for No.

Table S4. The Patient Education Materials Assessment Tool (PEMAT)

Intelligibility

| Item |  | Response Options | Rating |
| --- | --- | --- | --- |
| TOPIC: CONTENT | | | |
| 1 | The material makes its purpose completely evident. | Disagree=0, Agree=1 |  |
| TOPIC: WORD CHOICE & STYLE | | | |
| 3 | The material uses common, everyday language. | Disagree=0, Agree=1 |  |
| 4 | Medical terms are used only to familiarize audience with the terms. When used, medical terms are defined. | Disagree=0, Agree=1 |  |
| 5 | The material uses the active voice. | Disagree=0, Agree=1 |  |
| TOPIC: ORGANIZATION | | | |
| 8 | The material breaks or "chunks" information into short sections. | Disagree=0, Agree=1,  Very short material=N/A |  |
| 9 | The material’s sections have informative headers. | Disagree=0, Agree=1,  Very short material=N/A |  |
| 10 | The material presents information in a logical sequence. | Disagree=0, Agree=1 |  |
| 11 | The material provides a summary. | Disagree=0, Agree=1,  Very short material=N/A |  |
| TOPIC: LAYOUT & DESIGN | | | |
| 12 | The material uses visual cues (e.g., arrows, boxes, bullets, bold, larger font, highlighting) to draw attention to key points. | Disagree=0, Agree=1, Video=N/A |  |
| 13 | Text on the screen is easy to read. | Disagree=0, Agree=1,  No text or all text is narrated=N/A |  |
| 14 | The material allows the user to hear the words clearly (e.g., not too fast, not garbled). | Disagree=0, Agree=1,  No narration=N/A |  |
| TOPIC: USE OF VISUAL AIDS | | | |
| 18 | The material uses illustrations and photographs that are clear and uncluttered. | Disagree=0, Agree=1,  No visual aids=N/A |  |
| 19 | The material uses simple tables with short and clear row and column headings. | Disagree=0, Agree=1,  No tables=N/A |  |

Intelligibility Score (%) = (Total Points / Total Possible Points*100)

Operability

| Item |  | Response Options | Rating |
| --- | --- | --- | --- |
| 20 | The material clearly identifies at least one action the user can take. | Disagree=0, Agree=1 |  |
| 21 | The material addresses the user directly when describing actions. | Disagree=0, Agree=1 |  |
| 22 | The material breaks down any action into manageable, explicit steps. | Disagree=0, Agree=1 |  |
| 25 | The material explains how to use the charts, graphs, tables, or diagrams to take actions. | Disagree=0, Agree=1,  No charts, graphs, tables, diagrams=N/A |  |

Operability Score (%) = (Total Points / Total Possible Points*100)


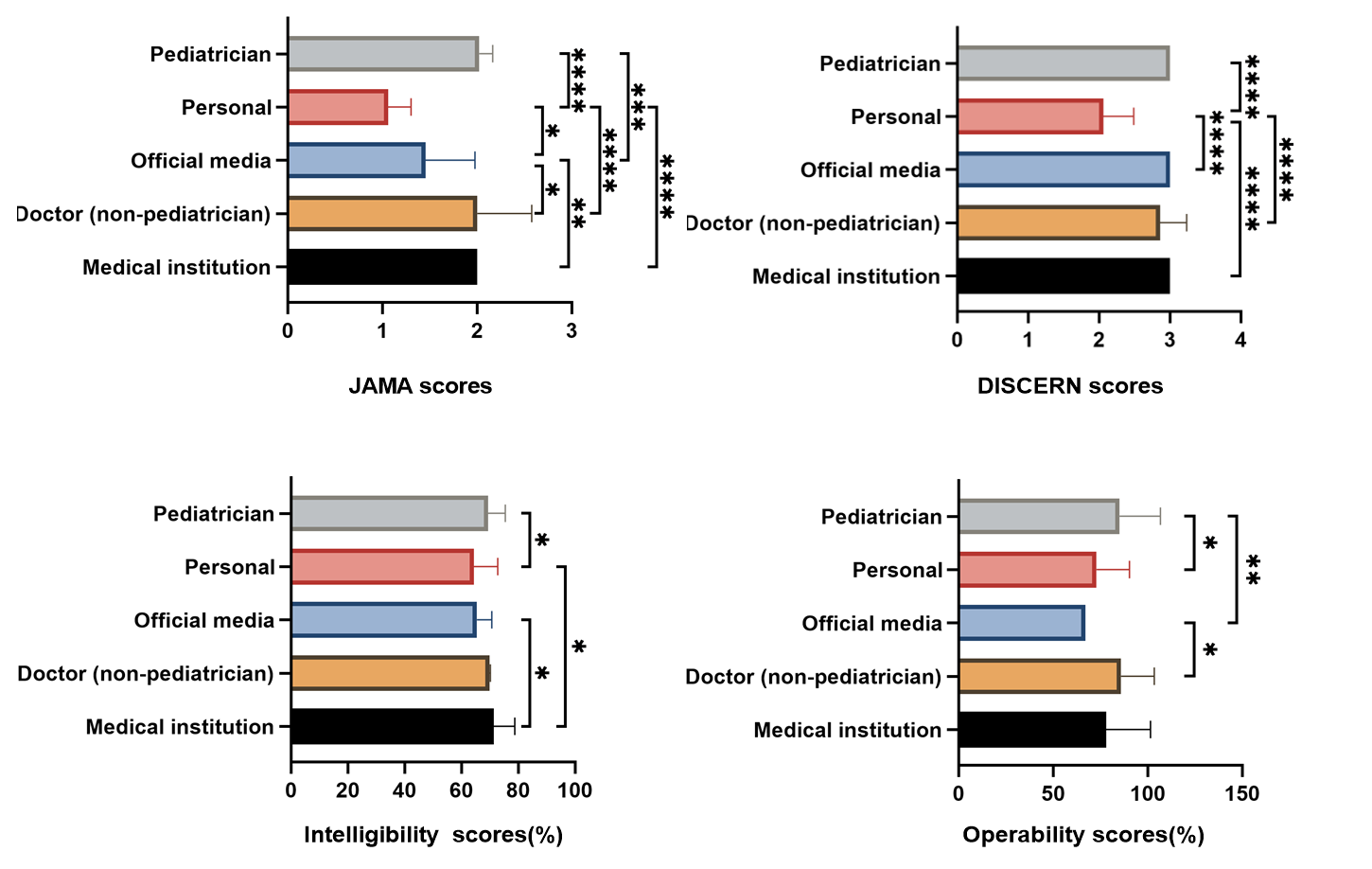


Figure S1 Quality of Videos Across Different Sources on Tiktok


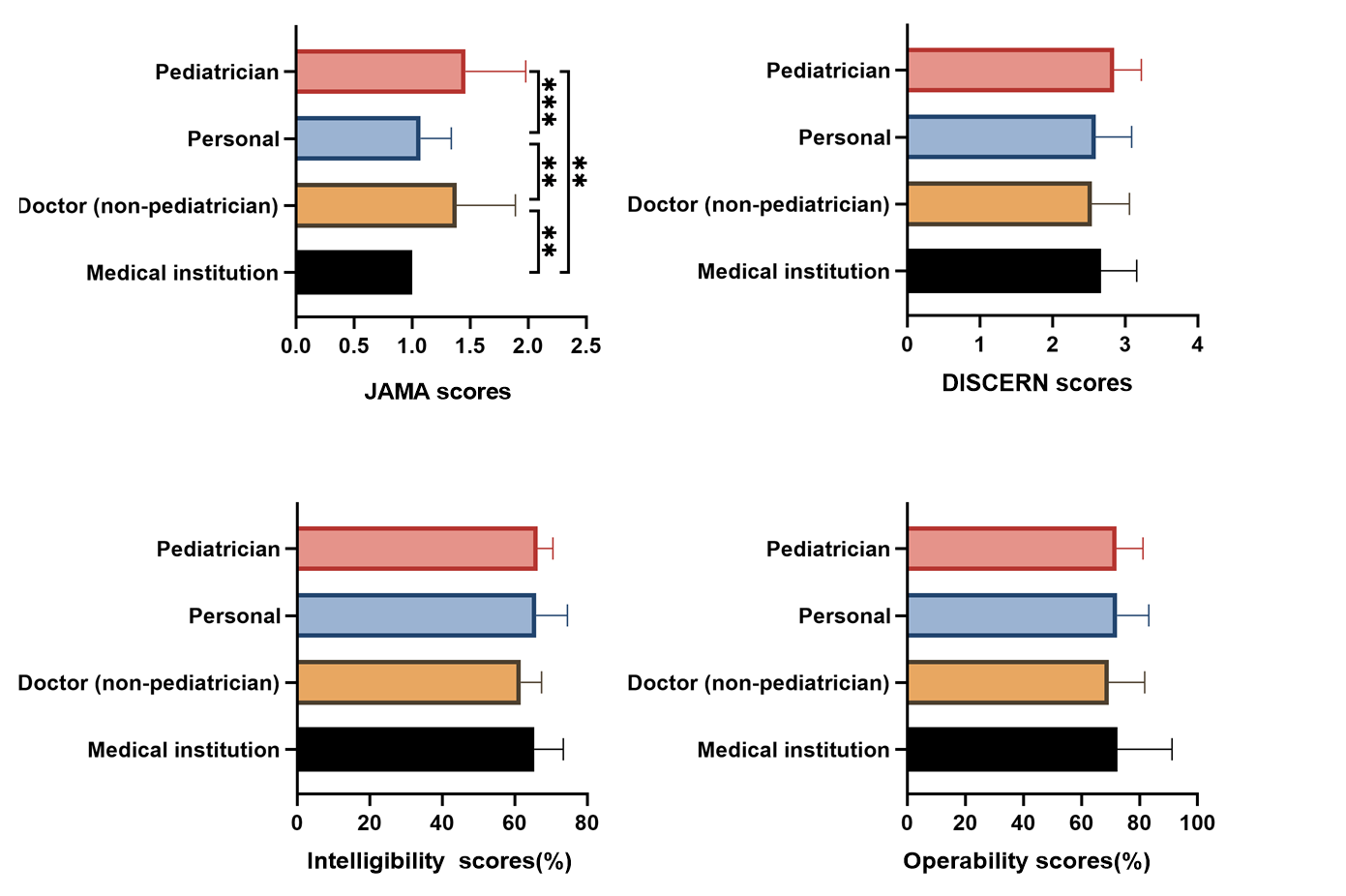


Figure S2 Quality of Videos Across Different Sources on Bilibili


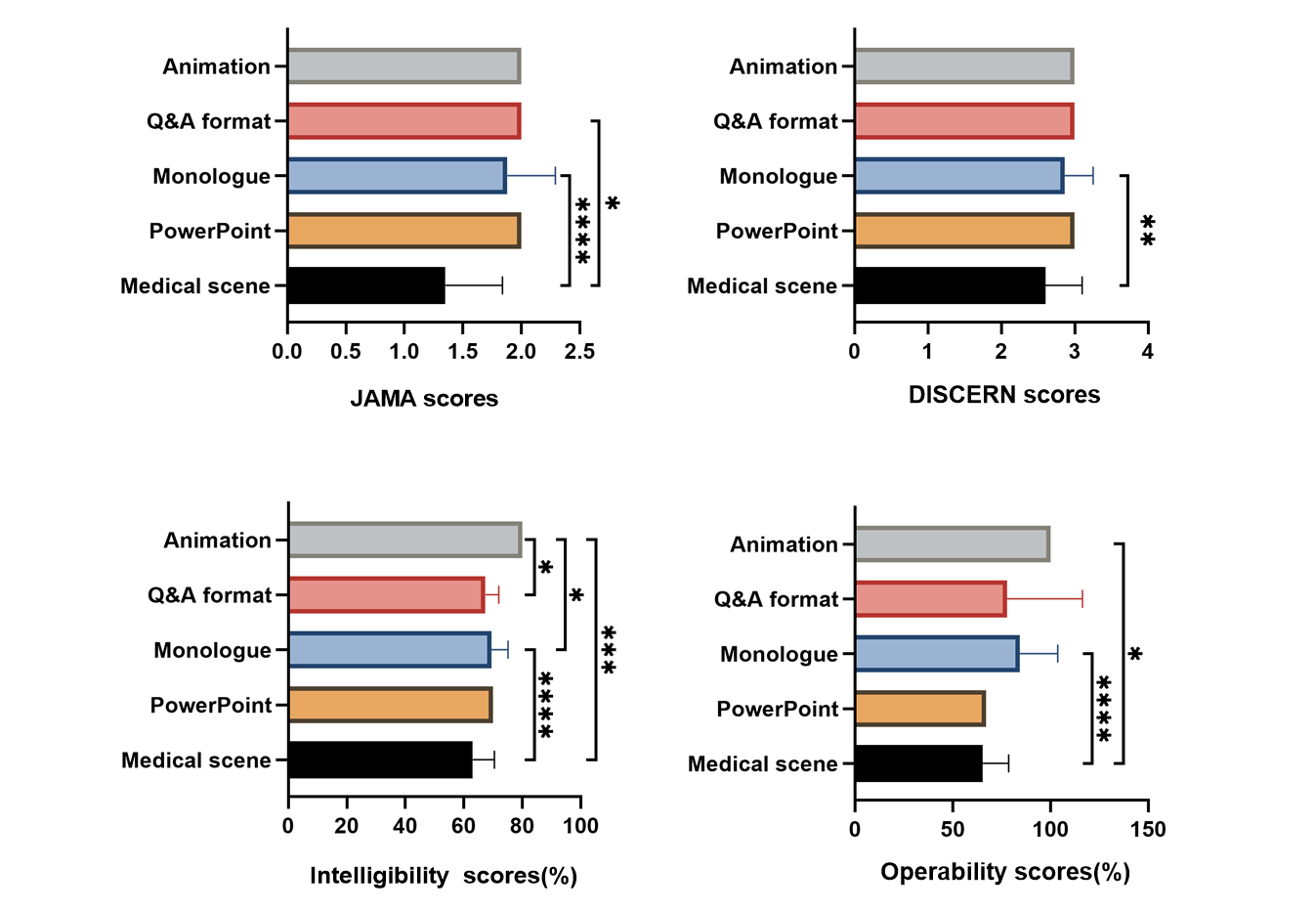


Figure S3 Quality of Videos Across Different Presentation Formats on Tiktok


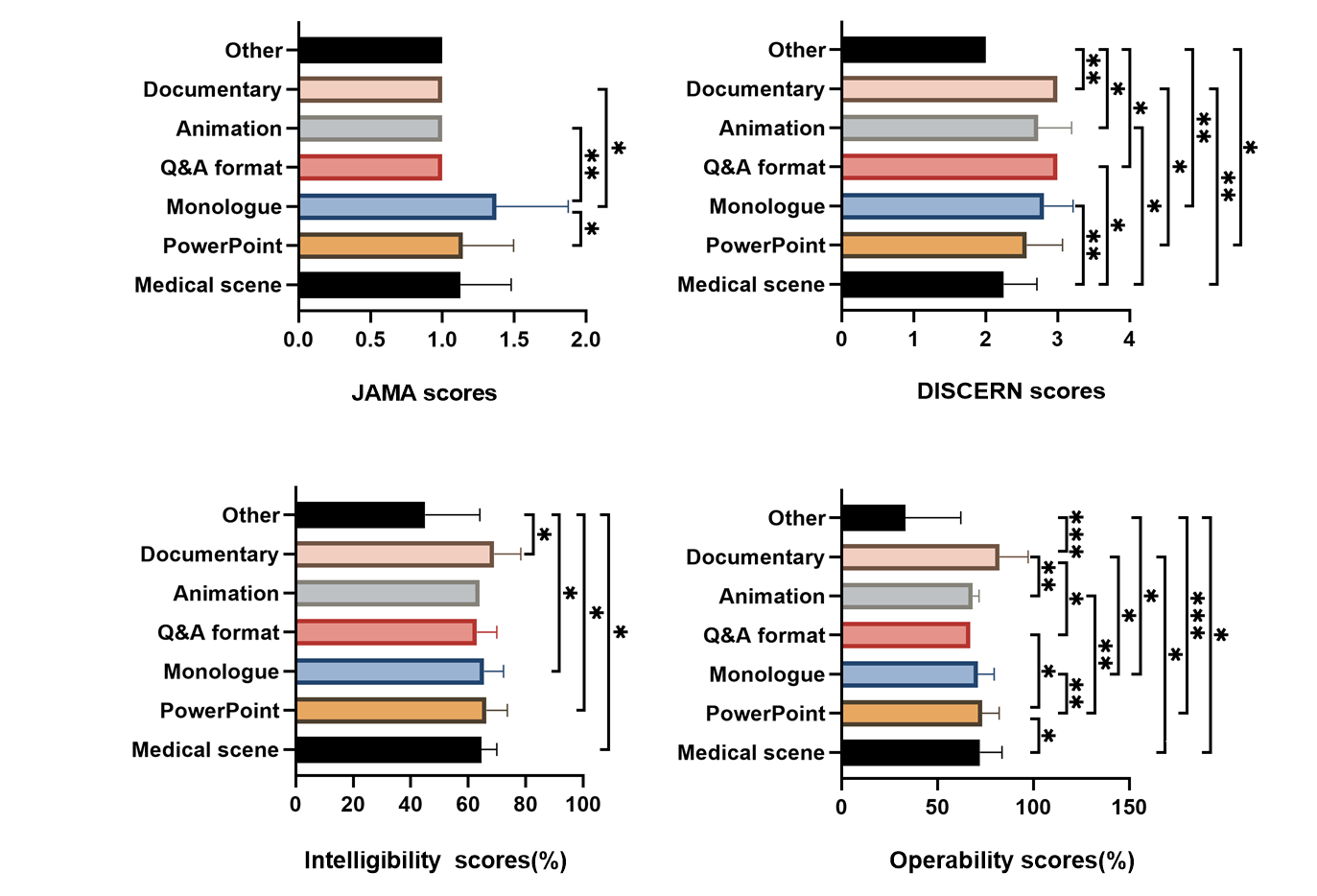


Figure S4 Quality of Videos Across Different Presentation Formats on Bilibili

SHAPE \* MERGEFORMAT
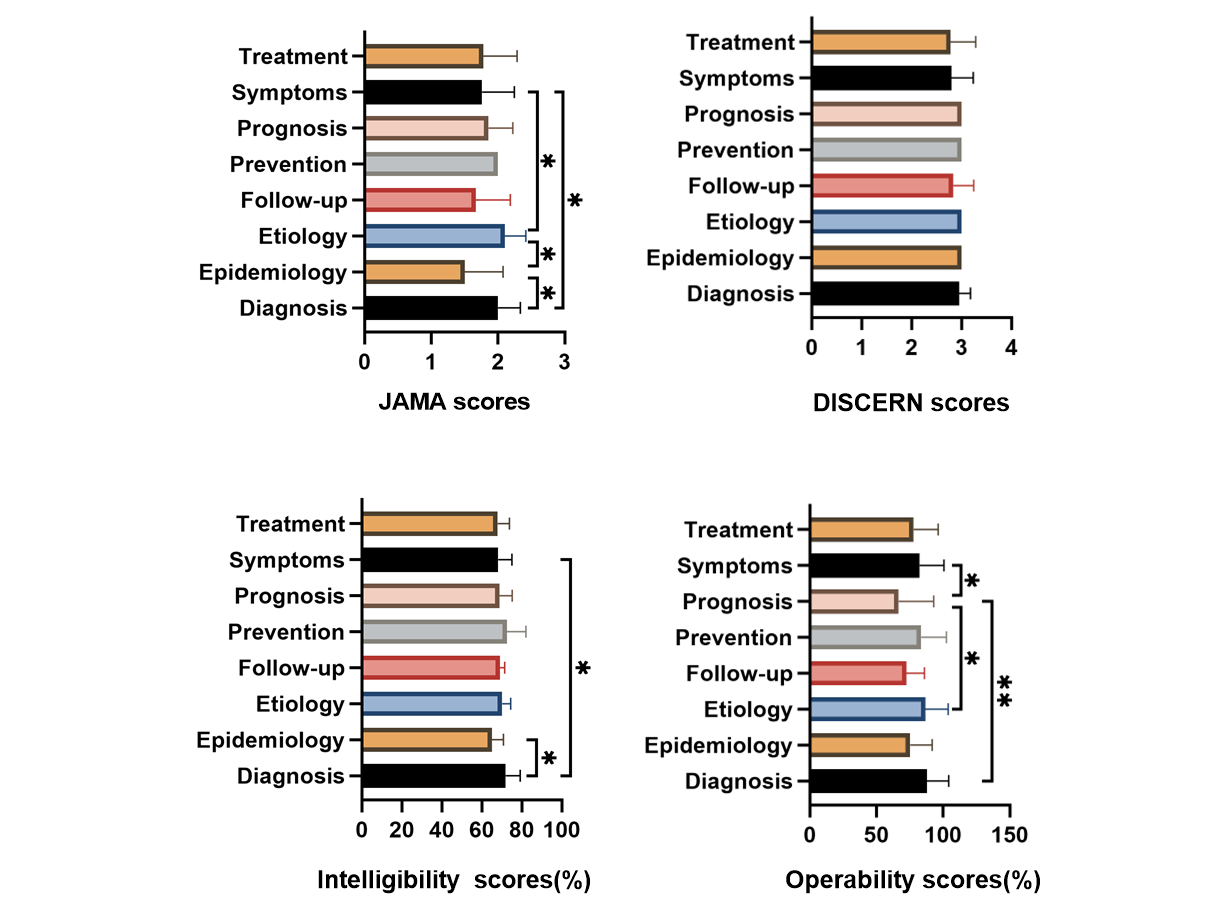


Figure S5 Quality of Videos Across Different Content Types on Tiktok


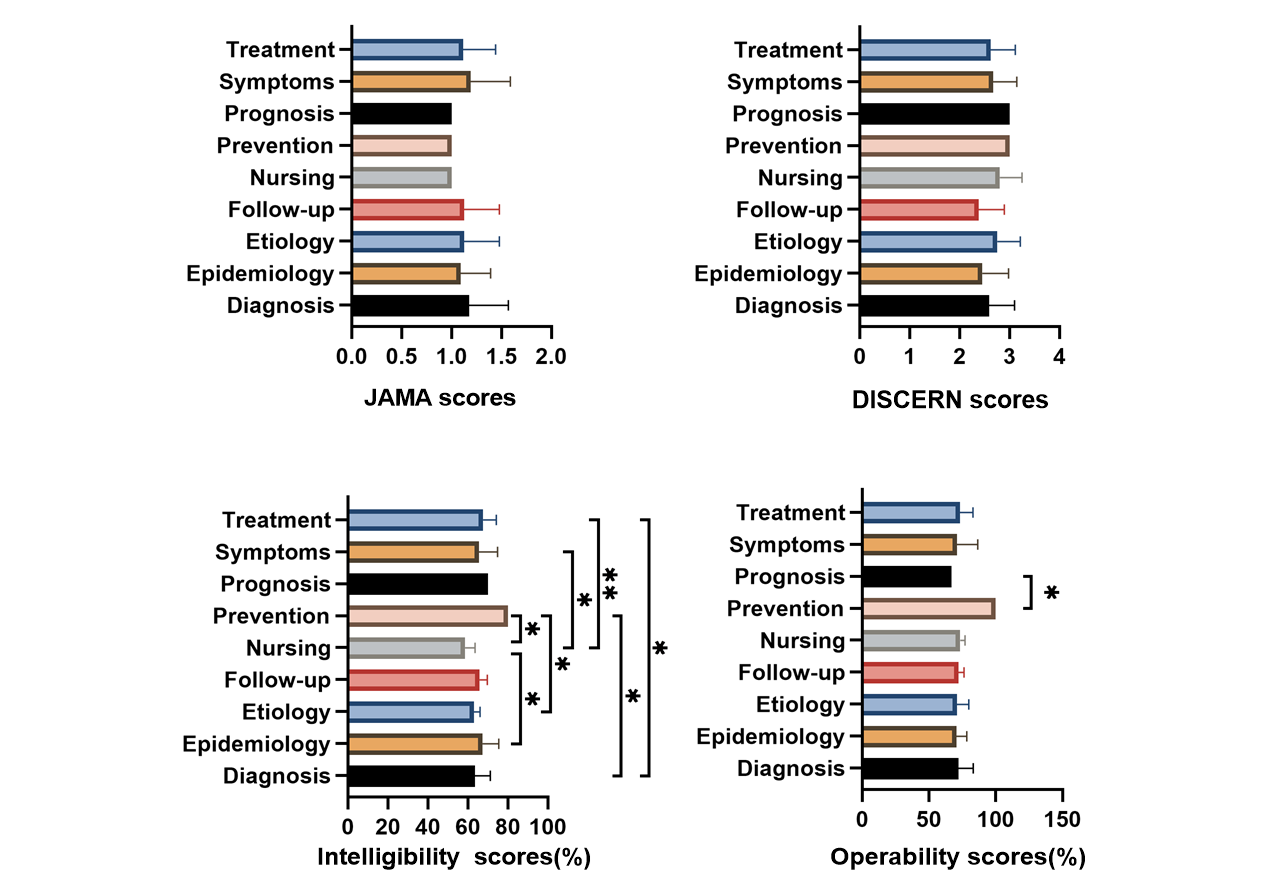


Figure S6 Quality of Videos Across Different Content Types on Bilibili

Table S4 Popularity of Videos Across Different Video Sources, Formats, and Content Types on Tiktok

| Variable | Likes, median (p25 - p75) | Comments, median (p25-p75) | Shares, median (p25 - p75) | Saves, median (p25 - p75) | Time, median (p25 - p75) |
| --- | --- | --- | --- | --- | --- |
| Video source (N=93) |  |  |  |  |  |
| Medical institution (n=9) | 355.00 (75.00 - 493.00) | 56.00 (3.00 - 83.00) | 144.00 (115.00 - 427.00) | 69.00 (37.00 - 112.00) | 77.00 (46.00 - 110.00) |
| Doctor (non-pediatrician) (n=7) | 1,956.00 (300.00 - 19,105.00) | 71.00 (6.00 - 1,770.00) | 235.00 (80.00 - 14,856.00) | 1,290.00 (84.00 - 9,662.00) | 86.00 (67.00 - 240.00) |
| Official media (n=11) | 200.00 (47.00 - 26,367.00) | 10.00 (1.00 - 2,051.00) | 246.00 (28.00 - 18,024.00) | 27.00 (5.00 - 11,785.00) | 27.00 (17.00 - 93.00) |
| Personal (n=17) | 268.00 (68.00 - 1,310.00) | 173.00 (42.00 - 333.00) | 104.00 (46.00 - 425.00) | 53.00 (13.00 - 222.00) | 128.00 (49.00 - 250.00) |
| Pediatrician (n=49) | 396.00 (96.00 - 2,137.00) | 63.00 (13.00 - 298.00) | 355.00 (58.00 - 1,216.00) | 156.00 (32.00 - 727.00) | 73.00 (49.00 - 114.00) |
| p-value | 0.281 | 0.446 | 0.571 | 0.231 | 0.06 |
| Video presentation format (N=93) |  |  |  |  |  |
| Animation (n=2) | 64.50 (20.00 - 109.00) | 1.50 (0.00 - 3.00) | 129.50 (115.00 - 144.00) | 43.50 (18.00 - 69.00) | 221.00 (111.00 - 331.00) |
| Medical scene (n=20) | 404.00 (68.00 - 1,841.50) | 147.00 (9.50 - 386.00) | 228.00 (53.50 - 1,249.00) | 72.50 (13.50 - 791.50) | 42.50 (21.50 - 94.00) |
| Monologue (n=67) | 396.00 (93.00 - 2,704.00) | 62.00 (16.00 - 312.00) | 306.00 (50.00 - 1,456.00) | 141.00 (28.00 - 831.00) | 86.00 (55.00 - 121.00) |
| PowerPoint (n=1) | 62.00 (62.00 - 62.00) | 1.00 (1.00 - 1.00) | 21.00 (21.00 - 21.00) | 46.00 (46.00 - 46.00) | 364.00 (364.00 - 364.00) |
| Q&A format (n=3) | 513.00 (55.00 - 545.00) | 38.00 (5.00 - 246.00) | 80.00 (46.00 - 1,036.00) | 85.00 (22.00 - 275.00) | 49.00 (46.00 - 246.00) |
| p-value | 0.417 | 0.11 | 0.606 | 0.749 | 0.014 |
| Video content (N=163) |  |  |  |  |  |
| Diagnosis (n=19) | 351.00 (96.00 - 2,137.00) | 61.00 (12.00 - 356.00) | 235.00 (35.00 - 839.00) | 137.00 (44.00 - 954.00) | 98.00 (65.00 - 132.00) |
| Epidemiology (n=4) | 599.00 (240.50 - 21,394.50) | 29.50 (14.00 - 6,552.50) | 604.00 (138.50 - 25,734.00) | 246.00 (26.00 - 6,115.00) | 70.50 (47.50 - 322.00) |
| Etiology (n=10) | 489.00 (281.00 - 2,035.00) | 64.50 (37.00 - 312.00) | 476.50 (195.00 - 1,456.00) | 206.00 (104.00 - 445.00) | 95.50 (55.00 - 113.00) |
| Follow-up (n=6) | 32.00 (23.00 - 68.00) | 3.00 (2.00 - 18.00) | 28.00 (25.00 - 31.00) | 11.50 (5.00 - 13.00) | 112.00 (93.00 - 321.00) |
| Prevention (n=4) | 212.00 (66.50 - 1,960.00) | 41.00 (2.50 - 460.00) | 177.00 (86.00 - 2,223.00) | 80.50 (36.50 - 910.00) | 132.50 (74.50 - 246.50) |
| Prognosis (n=14) | 186.00 (55.00 - 493.00) | 40.50 (3.00 - 172.00) | 133.00 (31.00 - 447.00) | 58.00 (22.00 - 112.00) | 58.00 (46.00 - 121.00) |
| Symptoms (n=79) | 417.00 (81.00 - 2,704.00) | 61.00 (12.00 - 364.00) | 266.00 (58.00 - 1,482.00) | 137.00 (27.00 - 831.00) | 79.00 (46.00 - 127.00) |
| Treatment (n=27) | 96.00 (55.00 - 603.00) | 27.00 (3.00 - 122.00) | 63.00 (28.00 - 417.00) | 44.00 (13.00 - 239.00) | 106.00 (53.00 - 194.00) |
| p-value | 0.015 | 0.092 | 0.024 | 0.019 | 0.377 |

Table S5 Popularity of Videos Across Different Video Sources, Formats, and Content Types on Bilibili

| Variable | Likes, median (p25 - p75) | Comments, median (p25-p75) | Shares, median (p25 - p75) | Saves, median (p25 - p75) | Time, median (p25 - p75) |
| --- | --- | --- | --- | --- | --- |
| Video source (N=53) |  |  |  |  |  |
| Medical institution (n=6) | 10.00 (0.00 - 29.00) | 0.00 (0.00 - 0.00) | 3.00 (1.00 - 24.00) | 7.00 (0.00 - 48.00) | 197.50 (169.00 - 563.00) |
| Doctor (non-pediatrician) (n=8) | 35.50 (10.50 - 62.50) | 2.00 (1.50 - 4.00) | 9.50 (3.50 - 18.50) | 22.50 (9.50 - 95.00) | 170.50 (114.00 - 710.50) |
| Personal (n=31) | 13.00 (6.00 - 88.00) | 1.00 (0.00 - 19.00) | 15.00 (5.00 - 44.00) | 28.00 (7.00 - 66.00) | 328.00 (202.00 - 1,552.00) |
| Pediatrician (n=8) | 14.00 (4.00 - 27.50) | 1.50 (0.50 - 2.50) | 15.00 (4.00 - 20.50) | 13.50 (3.50 - 28.50) | 245.00 (93.00 - 812.00) |
| p-value | 0.351 | 0.035 | 0.443 | 0.289 | 0.516 |
| Video presentation format (N=53) |  |  |  |  |  |
| Animation (n=4) | 101.50 (60.50 - 184.50) | 7.50 (2.50 - 15.00) | 92.00 (54.00 - 103.50) | 55.00 (36.50 - 135.00) | 779.50 (227.50 - 2,083.50) |
| Documentary (n=3) | 13.00 (7.00 - 3,505.00) | 1.00 (0.00 - 704.00) | 29.00 (15.00 - 523.00) | 16.00 (7.00 - 335.00) | 202.00 (51.00 - 3,721.00) |
| Medical scene (n=6) | 22.00 (6.00 - 130.00) | 3.00 (1.00 - 25.00) | 18.50 (2.00 - 27.00) | 12.50 (1.00 - 56.00) | 328.50 (41.00 - 547.00) |
| Other (n=2) | 304.00 (12.00 - 596.00) | 10.00 (0.00 - 20.00) | 9.00 (9.00 - 9.00) | 40.50 (28.00 - 53.00) | 282.00 (256.00 - 308.00) |
| Monologue (n=12) | 18.00 (3.00 - 35.50) | 2.00 (0.00 - 3.00) | 6.50 (2.00 - 12.00) | 14.00 (3.00 - 22.50) | 170.50 (93.00 - 540.00) |
| PowerPoint (n=24) | 13.50 (3.50 - 38.50) | 0.50 (0.00 - 2.00) | 15.00 (3.50 - 26.00) | 28.50 (7.00 - 58.50) | 525.00 (176.50 - 1,870.50) |
| Q&A format (n=2) | 2.00 (1.00 - 3.00) | 0.50 (0.00 - 1.00) | 1.00 (0.00 - 2.00) | 1.50 (0.00 - 3.00) | 144.50 (6.00 - 283.00) |
| p-value | 0.139 | 0.381 | 0.015 | 0.129 | 0.384 |
| Video content (N=119) |  |  |  |  |  |
| Diagnosis (n=17) | 27.00 (12.00 - 46.00) | 1.00 (0.00 - 3.00) | 15.00 (8.00 - 25.00) | 43.00 (22.00 - 86.00) | 762.00 (176.00 - 1,956.00) |
| Epidemiology (n=11) | 22.00 (6.00 - 88.00) | 1.00 (0.00 - 19.00) | 24.00 (8.00 - 54.00) | 46.00 (26.00 - 174.00) | 1,231.00 (78.00 - 2,164.00) |
| Etiology (n=8) | 59.50 (12.50 - 375.00) | 7.50 (0.00 - 23.00) | 37.00 (7.50 - 82.00) | 50.00 (23.00 - 252.00) | 1,272.50 (484.50 - 2,446.00) |
| Follow-up (n=8) | 6.50 (3.50 - 41.00) | 0.50 (0.00 - 4.00) | 11.50 (3.50 - 17.00) | 19.50 (5.00 - 39.00) | 2,175.00 (437.50 - 2,900.00) |
| Nursing (n=5) | 6.00 (2.00 - 8.00) | 0.00 (0.00 - 0.00) | 8.00 (4.00 - 13.00) | 16.00 (6.00 - 18.00) | 561.00 (207.00 - 646.00) |
| Prevention (n=1) | 13.00 (13.00 - 13.00) | 0.00 (0.00 - 0.00) | 29.00 (29.00 - 29.00) | 16.00 (16.00 - 16.00) | 202.00 (202.00 - 202.00) |
| Prognosis (n=1) | 8.00 (8.00 - 8.00) | 4.00 (4.00 - 4.00) | 2.00 (2.00 - 2.00) | 0.00 (0.00 - 0.00) | 41.00 (41.00 - 41.00) |
| Symptoms (n=42) | 19.50 (5.00 - 68.00) | 1.00 (0.00 - 8.00) | 15.00 (5.00 - 29.00) | 24.00 (6.00 - 53.00) | 291.00 (139.00 - 1,552.00) |
| Treatment (n=26) | 12.50 (7.00 - 31.00) | 1.00 (0.00 - 3.00) | 15.00 (8.00 - 24.00) | 25.50 (7.00 - 48.00) | 313.50 (186.00 - 1,552.00) |
| p-value | 0.406 | 0.506 | 0.482 | 0.059 | 0.157 |


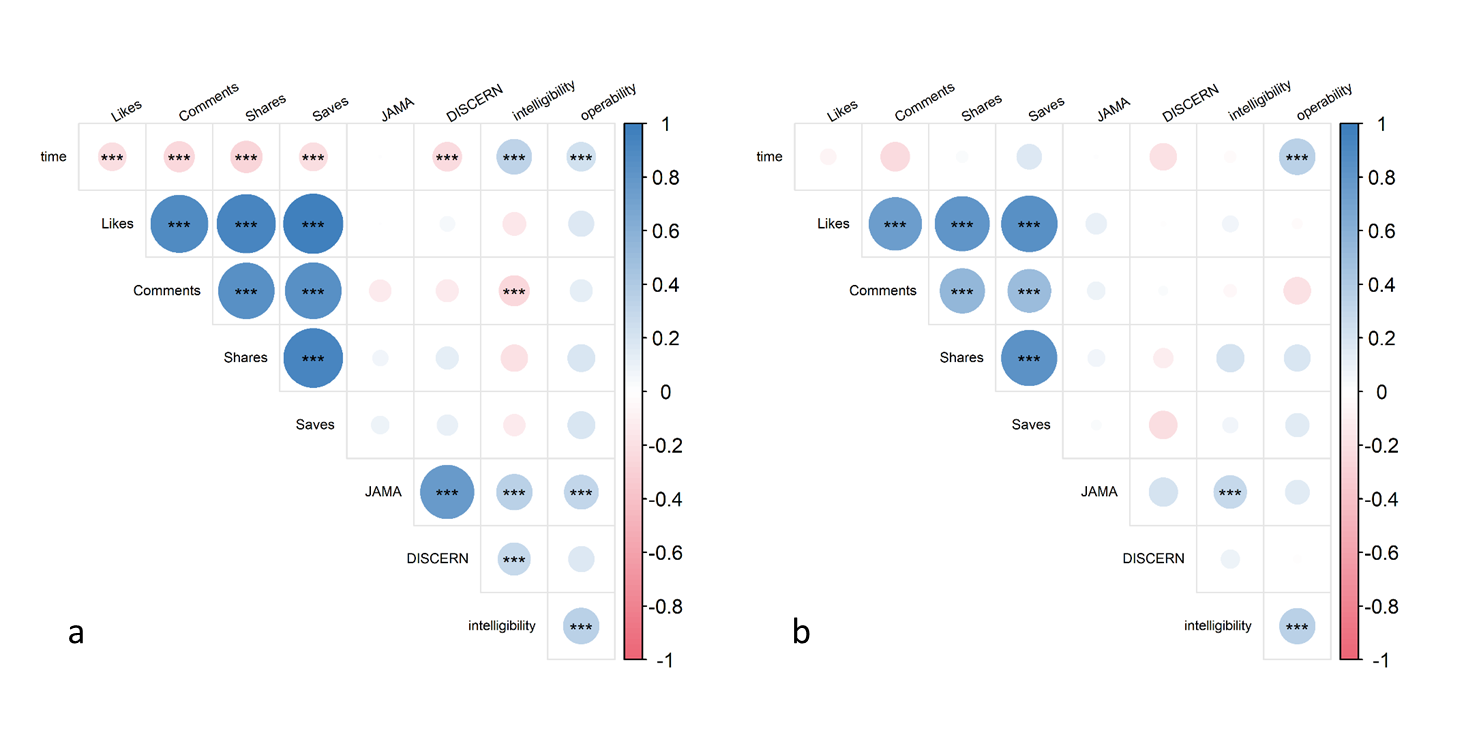


Figure S7 Correlation Analysis Between Video Variables and Video Quality on Each Platform (a: TikTok; b: Bilibili)
